# Leucine-rich pentatricopeptide repeat-containing protein (LRPPRC)-stabilized lncRNA small nucleolar RNA host gene 15 (*Snhg15*) modulates hematopoietic injury induced by γ-ray irradiation via m^6^A modification

**DOI:** 10.1186/s43556-025-00279-2

**Published:** 2025-06-25

**Authors:** Shuqin Zhang, Yajia Cheng, Yujia Gao, Feifei Xu, Yuna Wang, Junling Zhang, Yue Shang, Deguan Li, Saijun Fan

**Affiliations:** State Key Laboratory of Advanced Medical Materials and Devices, Tianjin Key Laboratory of Radiation Medicine and Molecular Nuclear Medicine, Tianjin Institutes of Health Science, Institute of Radiation Medicine, Chinese Academy of Medical Sciences & Peking Union Medical College, Tianjin, 300192 People’s Republic of China

**Keywords:** Radiation injury, RNA methylation, Small nucleolar RNA host gene 15 (*Snhg15*), Leucine-rich pentatricopeptide repeat-containing protein (LRPPRC), Hematopoiesis

## Abstract

With advancements in radiotherapy technologies, the detrimental effects of ionizing radiation on biological systems, particularly the hematopoietic system, have caused significant concern. N^6^-methyladenosine (m^6^A), the most pervasive representative of post-transcriptional modifications, plays critical roles in diverse biological events. Non-coding RNA comprises the vast majority of the human genome. This study aimed to explore the role of long non-coding RNA (lncRNA) m^6^A modification in γ-ray irradiation-induced hematopoietic injury. By using mouse models, it was found that γ-radiation rapidly damaged hematopoietic bone marrow cells (BMCs), triggering apoptosis, oxidative stress and DNA damage, along with up-regulation of m^6^A Reader proteins. We revealed the time-conditioned landscape of lncRNA m^6^A methylome of BMCs in the short term after radiation and found that a dynamic “change-then-recover” trend involved. LncRNA *Snhg15* was identified as a key regulator through integration analysis of the methylome and transcriptome data. Its m^6^A modification was closely related to progression of radiation injury in BMCs. Further research demonstrated that the novel m^6^A Reader LRPPRC could interact with the modification site of *Snhg15*, stabilize *Snhg15* and promote its expression, thereby exacerbating radiation-induced injury to BMCs both in vitro and in vivo. Knockdown of *Lrpprc* or *Snhg15* could alleviate the radiation injury to the hematopoietic system. Additionally, the LRPPRC-*Snhg15* axis was involved in the radio-protective efficacy of gut microbiota-derived valeric acid. These findings uncover a novel mechanism by which m^6^A-modified lncRNA *Snhg15* is stabilized by LRPPRC modulates γ-irradiation-induced hematopoietic injury, providing potential therapeutic targets for the prevention and treatment of radiation injuries.

**Supplementary Information:**

The online version contains supplementary material available at 10.1186/s43556-025-00279-2.

## Introduction

Radiotherapy remains a cornerstone in the treatment of various malignancies [1, 2]. While advancements in image-guided radiotherapy (IGRT) and proton therapy have improved cancer management, radiation-induced hematopoietic injury persists as a dose-limiting toxicity in patients [3]. Ionizing radiation (IR) exerts cytotoxic effects on bone marrow hematopoietic stem cells (HSCs), leading to hematopoietic dysfunction and microenvironmental disruption [4]. Although direct DNA damage and free radical-mediated genotoxicity are well characterized, the transcriptional and post-transcriptional regulatory mechanisms underlying radiation responses remain incompletely understood [5, 6]. Elucidating these pathways is critical for developing radio-protective strategies and improving precision radiotherapy safety.

N^6^-methyladenosine (m^6^A), the most abundant post-transcriptional RNA modification, has emerged as a key regulator of cellular responses to stress [7]. This modification is conserved across eukaryotes, prokaryotes, and viruses, with preferential enrichment in RRACH ([G > A]m^6^AC[U > A > C]) motifs near stop codons and 3'-untranslated regions (3'-UTRs) [8, 9]. m^6^A dynamics are orchestrated by “Writers” (methyltransferases), “Erasers” (demethylases) and “Readers” (m^6^A-binding proteins). The writer-eraser-reader machinery orchestrates various life processes through RNA modification-mediated control of RNA fate, precisely regulating the lifespan, translation and subcellular localization of related transcripts [10]. Classic Readers include YTH domain proteins, heterogeneous nuclear ribonucleoproteins (HNRNPs), and IGF2 mRNA-binding proteins (IGF2BPs) [11, 12]. METTL3 also serves as a reader and interacts with the eukaryotic translation initiation factor 3 subunit h (eIF3h) to promote mRNA translation [13]. These recognition proteins specifically recognize and bind to m^6^A-modified RNAs, thereby ultimately affecting the fate of the modified-RNAs and mediating related biological functions [14]. The Leucine-rich pentatricopeptide repeat-containing protein (LRPPRC), initially identified as a protein involved in mitochondrial function and energy metabolism [15], has recently been reported to function as an m^6^A Reader. It can affect the stability, nuclear export and serve as an immune marker by binding to m^6^A-modified mRNAs [16–19].

Long non-coding RNAs (LncRNAs), a major class of non-coding RNAs, play pivotal roles in gene regulation, cell differentiation, and disease pathogenesis [20]. m^6^A modification of lncRNAs modulates their cleavage, translocation and stability [21]. For example, METTL3-mediated m^6^A modification of lncRNA *MEG3* regulates radiation-induced liver injury via YTH domain containing 1 (YTHDC1)-dependent stabilization [22]. However, the role of lncRNA m^6^A modification in hematopoietic radiation injury remains unclear. LncRNA small nucleolar RNA host gene 15 (*SNHG15*) has been implicated in tumorigenesis and diverse injury phenotypes, including cerebral ischemia–reperfusion injury and skeletal muscle injury [23–26]. However, its m^6^A-dependent function in γ-irradiation-induced hematopoietic injury is unknown.

To this end, we aimed to interrogate the immediate effect of γ-irradiation on hematopoietic bone marrow cells (BMCs), uncover the time-conditioned panorama of lncRNA m^6^A methylome and clarify the lncRNA m^6^A-related mechanisms underlying the hematopoietic radiation injury. We found that 4 Gy of total body irradiation (TBI) by γ-rays dampened BMCs rapidly in mouse, methylated (m^6^A) RNA immunoprecipitation with high-throughput sequencing (MeRIP-seq) revealed that a dynamic lncRNA m^6^A methylome with alterations of a “change-then-recover” trend was involved. Mechanistically, integration analysis of the MeRIP-seq and transcriptomic data and experimental methods demonstrated that LRPPRC stabilized lncRNA *Snhg15* in an m^6^A-dependent fashion, promoted its expression and exacerbated the hematopoietic radiation injury in vitro and in vivo. The LRPPRC-*Snhg15* axis was also implicated in the radio-protective efficacy of gut microbiota-derived valeric acid (VA). Our findings not only highlight the significance of lncRNA m^6^A modification in radiobiology, but also provide potential m^6^A-based targets and inspire new ideas of developing novel agents for radiation protection.

## Results

### Rapid damage of bone marrow hematopoietic cells by γ-ray irradiation involves altered expression of m^6^A Readers

**Fig. 1:**
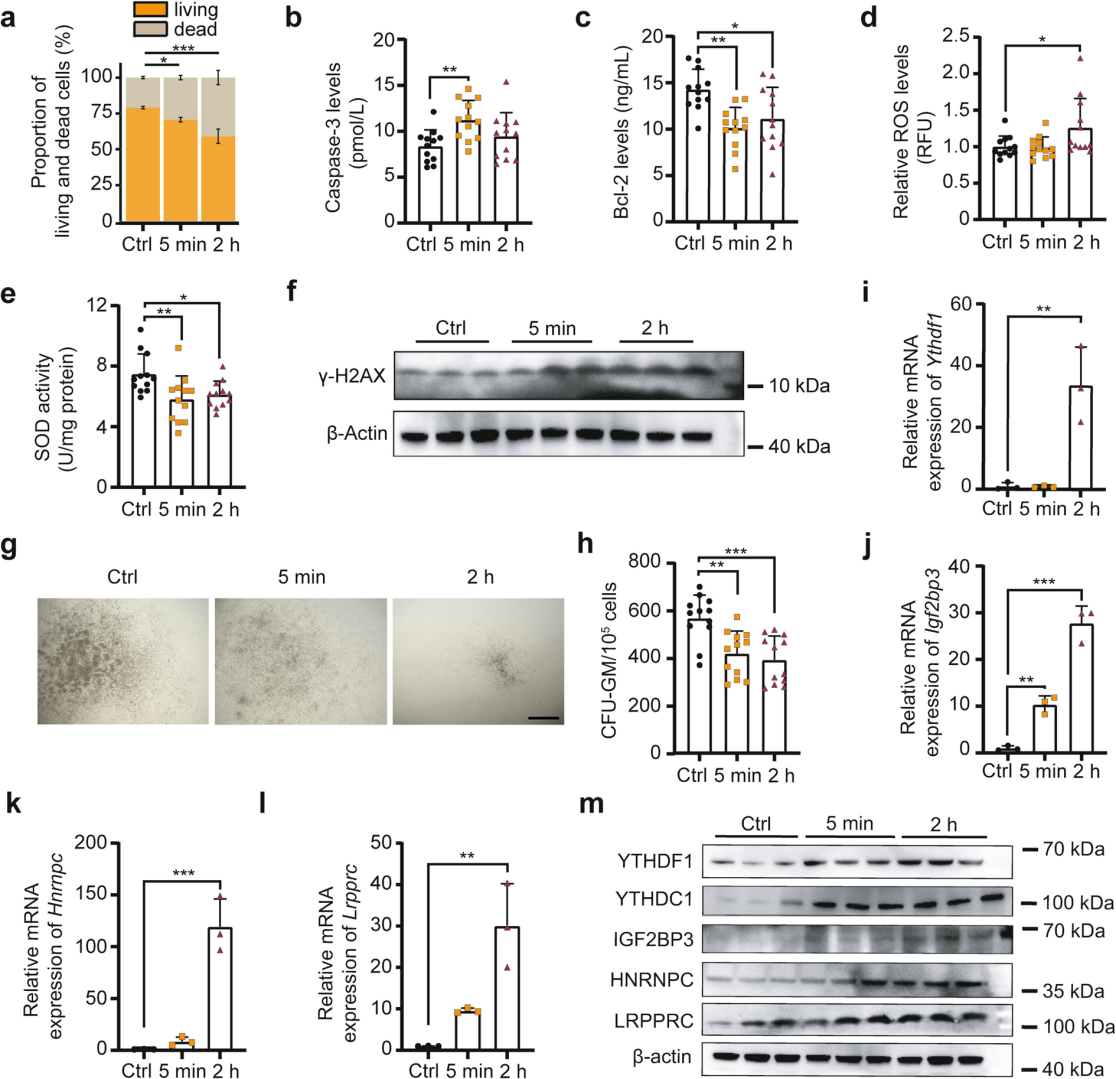
Rapid damage of bone marrow hematopoietic cells by γ-ray irradiation involves altered expression of m^6^A Readers. **a** Statistical analysis of viable and dead BMC proportions in Ctrl, 5 min and 2 h groups. (*n* = 3 biological replicates; each replicate pooled BMCs from 4 mice). **b** and** c** ELISA quantification of pro-apoptotic Caspase-3 (**b**) and anti-apoptotic Bcl-2 (**c**) levels in BMCs. **d** and** e** Oxidative stress markers: intracellular ROS levels (**d**) and SOD activity (**e**). **f** Western blot analysis of γ-H2AX protein expression. Each biological replicate pooled BMCs from 4 mice. **g** and **h** Representative images and count statistics of CFU-GM from Ctrl, 5 min and 2 h groups. Scale bar: 500 μm. **i-l** QRT-PCR analysis of m^6^A Reader mRNA expression: *Ythdf1* (**i**), *Igf2bp3* (**j**), *Hnrnpc* (**k**) and *Lrpprc* (**l**). Each biological replicate pooled BMCs from 4 mice. **m** Western blot analysis of m^6^A Reader protein expression. Each biological replicate pooled BMCs from 4 mice. Data are presented as mean ± SD. Statistical significance was determined by one-way ANOVA: *P < 0.05, **P < 0.01, ***P < 0.001

To investigate the immediate effects of ionizing radiation on hematopoietic organs, BMCs were collected at 0 min, 5 min, and 2 h post-4 Gy TBI with γ-rays to examine injury phenotypes. The proportion of viable hematopoietic cells decreased, while dead cells increased, as early as 5 min post-irradiation (Fig. 1a). Morphological and size alterations in cells were observed at 2 h (Fig. S1a and b). The Level of the pro-apoptotic factor Cysteine aspartate-specific proteinase-3 (Caspase-3) increased rapidly following irradiation, whereas that of the anti-apoptotic factor B-cell lymphoma/Leukemia-2 (Bcl-2) decreased (Fig. 1b and c). Oxidative stress markers reactive oxygen species (ROS) and superoxide dismutase (SOD) mirrored Caspase-3 and Bcl-2 trends (Fig. 1d and e). Γ-H2 AX, a marker of DNA double-strand breaks, increased time-dependently in BMCs post-irradiation (Fig. 1f). These results indicate that γ-ray exposure induces apoptosis, oxidative stress and DNA damage rapidly in BMCs. Additionally, colony-forming unit-granulocyte macrophage (CFU-GM) formation in 5 min and 2 h groups was significantly reduced compared to controls, confirming impaired hematopoietic progenitor cell (HPC) viability after irradiation (Fig. 1g and h).

Given the critical roles of m^6^A modification in biological processes and the functional importance of Reader proteins in mediating m^6^A effects [14], we measured expression of representative m^6^A Readers in BMCs post-irradiation. Notably, both mRNA and protein levels of YTH domain-containing family protein 1 (*Ythdf1*), *Ythdc1*, *Igf2bp3*, *Hnrnpc*, and *Lrpprc* increased within 2 h (Fig. 1i-m and Fig. S1c), suggesting m^6^A modification may contribute to acute radiation injury. Collectively, γ-ray exposure rapidly damages bone marrow hematopoietic cells, accompanied by upregulation of m^6^A Reader proteins.

### Presentation of the time-dependent m^6^A methylome of lncRNAs in BMCs following γ-irradiation

**Fig. 2:**
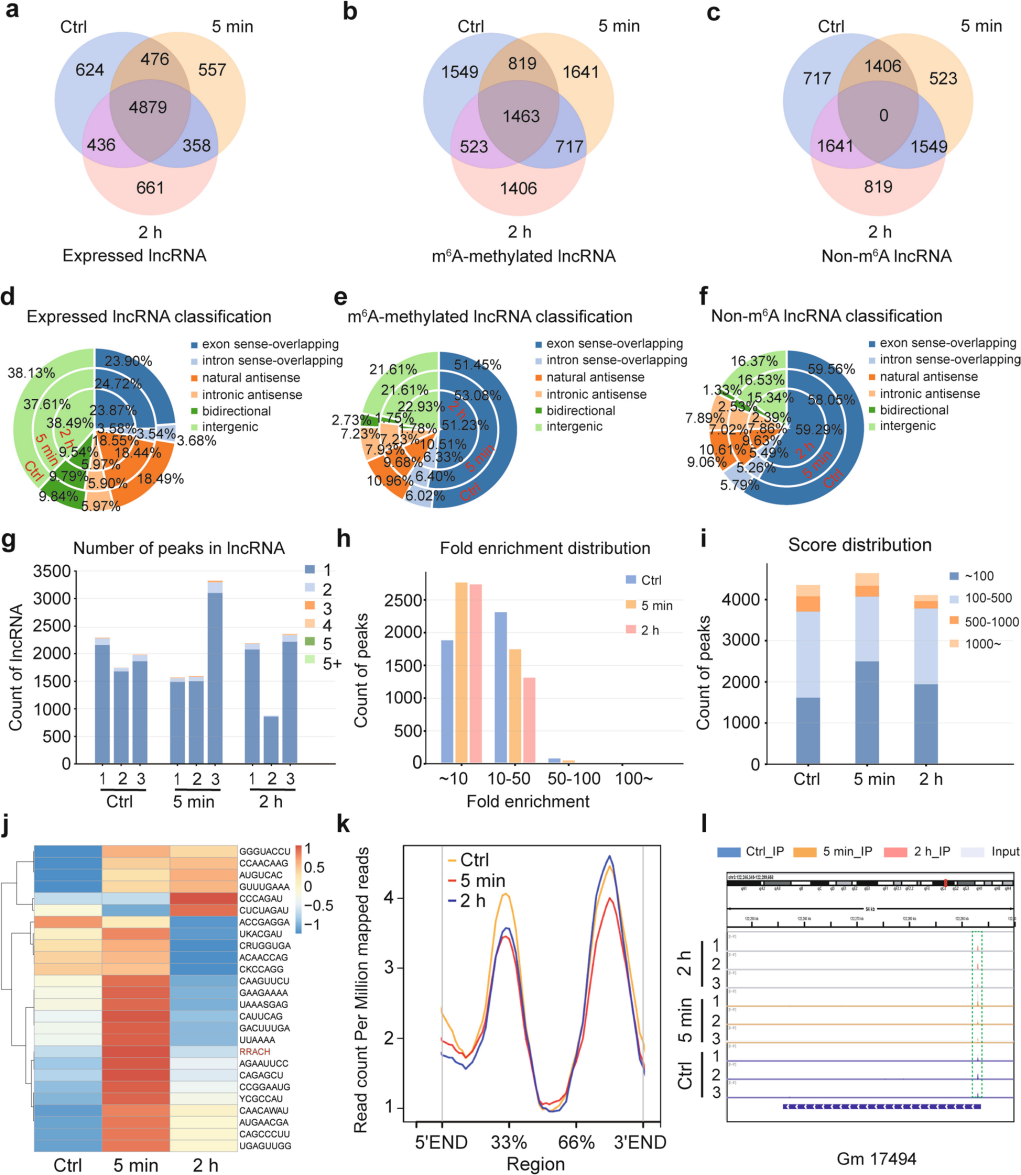
Presentation of the time-dependent m^6^A methylome of lncRNAs in BMCs following γ-irradiation. **a**-**c** The Venn Diagrams showing the numbers of the expressed lncRNAs (**a**), m^6^A-methylated lncRNAs (**b**) and unmethylted lncRNAs (**c**) in bone marrow samples of Ctrl, 5 min and 2 h groups. **d-f** The classification and proportional statistics of lncRNAs with different origins at 0 min, 5 min and 2 h after 4 Gy γ-irradiation, under the three different modification states: the expressed lncRNAs (**d**), m^6^A-methylated lncRNAs (**e**) and non-m^6^A lncRNAs (**f**). **g** Counts of lncRNAs with different numbers of m^6^A peaks in each replicate of the three groups. **h** The specific numbers of m^6^A summits with distinct enrichment-folds for the three groups. **i** The quantitative distribution of m^6^A peaks across different enrichment score segments in each group. **j** Heatmap presenting the enrichment of each m^6^A motif in Ctrl, 5 min and 2 h groups, with the classic consensus motif of RRACH highlighted. **k** Curves visualizing the distribution mode of m^6^A modification along the whole lncRNA bodies. **l** IGV software displaying the representative of conserved m^6^A peaks among three time points

To uncover the time-dependent profile of m^6^A methylome in hematopoietic radiation injury, BMCs from mice euthanized at three post-irradiation time points were collected for RNA extraction, MeRIP-seq, and RNA-seq analyses. This study specifically investigated m^6^A methylomic and transcriptomic landscapes of lncRNAs. Venn diagrams revealed 6,415, 6,270, and 6,334 expressed lncRNAs in Ctrl, 5 min, and 2 h groups, respectively (transcriptomic data). The data showed a slight “down-then-up” trend, exemplified by unique transcripts per group (624 in Ctrl, 557 in 5 min, 661 in 2 h; Fig. 2a). MeRIP-seq identified 4,354 (Ctrl), 4,640 (5 min), and 4,109 (2 h) m^6^A-methylated lncRNAs, displaying an “up-then-down” pattern with distinct unique peaks (1,549 in Ctrl, 1,641 in 5 min, 1,406 in 2 h; Fig. 2b). Non-m^6^A lncRNA counts (3,818 in Ctrl, 3,478 in 5 min, 4,009 in 2 h) followed a “down-then-up” trajectory (Fig. 2c). These results validate the dynamic/reversible nature of m^6^A modification [10, 27] and suggest radiation acutely induces transient m^6^A fluctuations.

LncRNAs were classified into six categories (exon sense-overlapping, intron sense-overlapping, natural antisense, intronic antisense, bidirectional and intergenic) to analyze compositional shifts. Pie charts showed exon sense-overlapping and intergenic lncRNAs dominated expressed/methylated/non-methylated fractions. Most categories exhibited a “change-then-recover” dynamic pattern (Fig. 2d-f). Single-methylation events prevailed in all samples (Fig. 2g). Quantification of enrichment-fold distributions revealed ~ 50-fold peaks dominated, with ~ tenfold peaks increasing and > tenfold peaks decreasing post-irradiation (Fig. 2h). Methylation scores were mostly ~ 500, with ~ 100-score peaks showing “up-then-down” trends and 100–500-score peaks showing “down-then-up” trends (Fig. 2i). Motif analysis identified 26 prevalent m^6^A motifs, with most (including the canonical RRACH motif) showing increased methylation at 5 min and decreased at 2 h (Fig. 2j). Metagene analysis confirmed “change-then-recover” methylation patterns along lncRNA regions, with highest levels at 33% and 66%-3'END (Fig. 2k). Despite global dynamics, some peaks remained stable (e.g., Gm 17494; Fig. 2l), suggesting radiation-insensitive sites. In summary, we reveal the time-conditioned lncRNA m^6^A landscape with a ubiquitous “change-then-recover” fashion in the γ-radiation-evoked hematopoietic injury of BMCs.

### Differentially modified m^6^A sites on lncRNAs between distinct moments

**Fig. 3:**
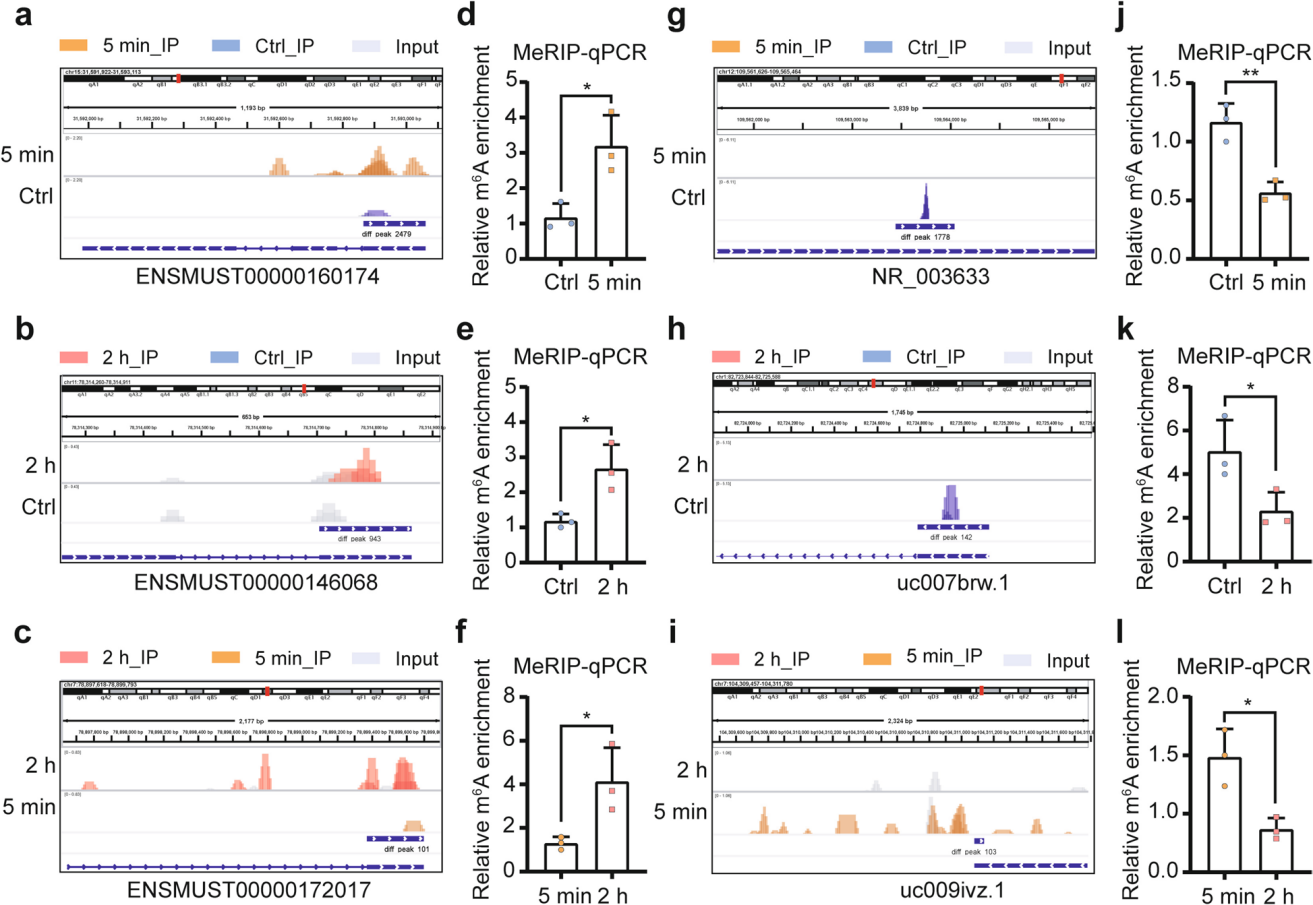
Differentially modified m^6^A sites on lncRNAs between distinct moments. **a**-**c** The representative up-regulated m^6^A peaks on lncRNAs of ENSMUST00000160174 (**a**), ENSMUST00000146068 (**b**) and ENSMUST00000172017 (**c**) in comparisons of 5 min vs Ctrl, 2 h vs Ctrl and 2 h vs 5 min were shown by IGV software, respectively. **d**-**f** The modification alterations of the above up-regulated peaks were validated by MeRIP-qPCR analysis. Each biological replicate pooled BMCs from 4 mice. **g**-**i** The representative down-regulated m^6^A peaks on lncRNAs of NR_003633 (**g**), uc007brw.1 (**h**) and uc009ivz.1 (**i**) in comparisons of 5 min vs Ctrl, 2 h vs Ctrl and 2 h vs 5 min were shown by IGV software, respectively. **j**-**l** The modification alterations of the above down-regulated peaks were validated by MeRIP-qPCR analysis. Each biological replicate pooled BMCs from 4 mice. Data are shown as the mean ± SD of three independent experiments. Statistical significance was determined by Student’s *t*-test: *, *P* < 0.05; **, *P* < 0.01

To explore the detailed changes of lncRNA m^6^A modification following radiation, we screened the differentially methylated m^6^A peaks on lncRNAs between each two cohorts based on the value of reads per million (RPM). 1501 up/877 down peaks in comparison of 5 min vs Ctrl, 884 up/993 down peaks in comparison of 2 h vs Ctrl and 10 up/13 down peaks in comparison of 2 h vs 5 min were identified, respectively. The representive m^6^A peaks in each comparison were selected by referring to parameters of enrichment score, fold-change and confidence degree of lncRNAs. For the up-regulated modifications, they were ENSMUST00000160174 (up_5 min vs Ctrl), ENSMUST00000146068 (up_2 h vs Ctrl) and ENSMUST00000172017 (up_2 h vs 5 min), respectively. Details of modifications at these sites were presented by IGV software (Fig. 3a-c). And the modification tendencies of these sites were also verified by MeRIP-qPCR (Fig. 3d-f). For the down-regulated modifications, they were NR_003633 (down_5 min vs Ctrl), uc007brw.1 (down_2 h vs Ctrl) and uc009ivz.1 (down_2 h vs 5 min), respectively. Details of modifications at these sites were also presented by IGV software (Fig. 3g-i), and MeRIP-qPCR was conducted to validate the modification tendencies of these sites (Fig. 3j-l). These findings imply the sensitivity of lncRNA m^6^A modification in response to radiation stimulation.

### Identification of m^6^A peaks with different trends of lncRNAs after radiation

**Fig. 4:**
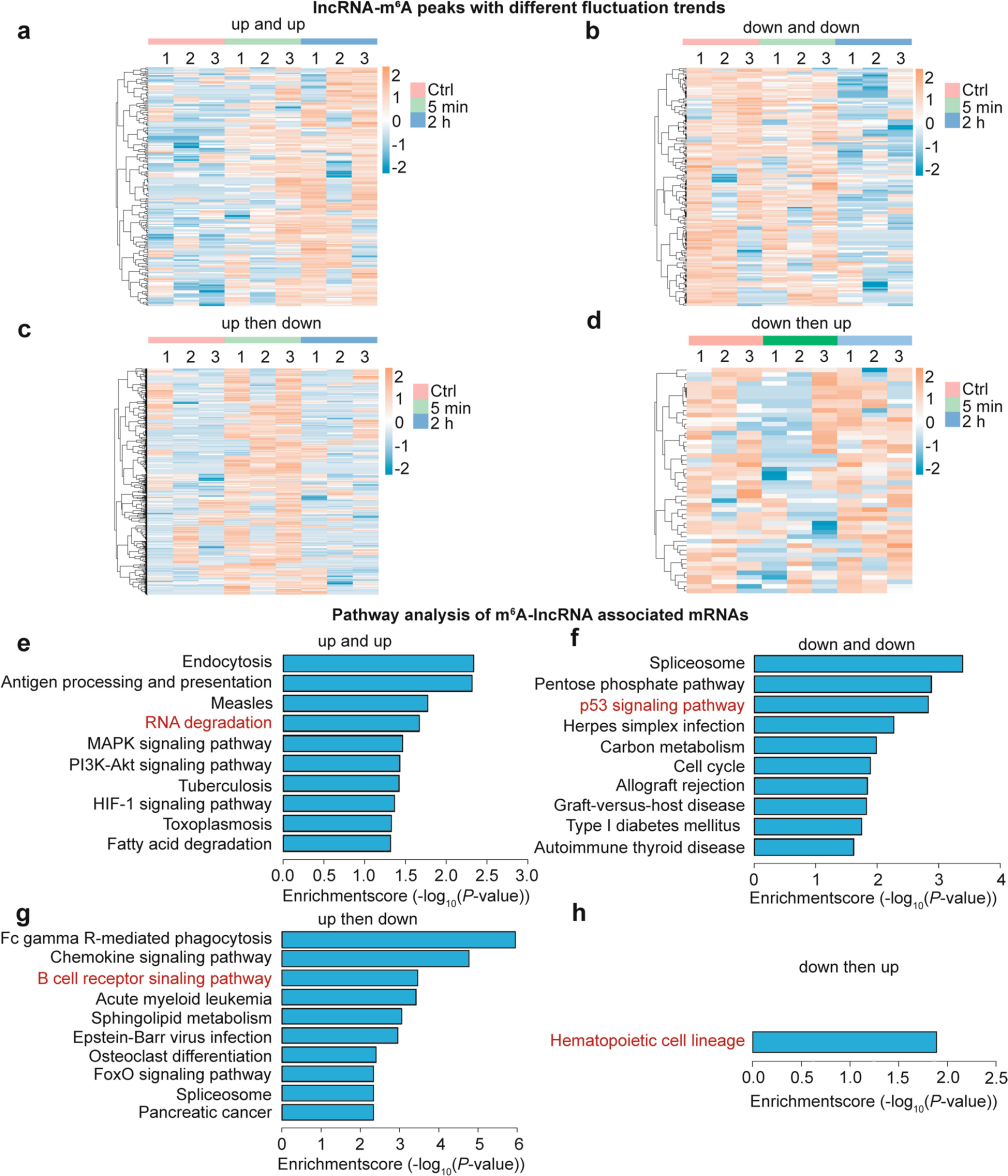
Identification of m^6^A peaks with different trends of lncRNAs after radiation. **a**-**d** The heatmaps showing the continuously regulated (up and up (**a**) and down and down (**b**)) and reversibly regulated (up then down (**c**) and down then up (**d**)) m^6^A summits of lncRNAs across Ctrl, 5 min and 2 h groups. **e-h** KEGG pathway analyses of mRNAs associated with m^6^A-methylated lncRNAs with the peaks that were continuously regulated (up and up (**e**) and down and down (**f**)) or reversibly regulated (up then down (**g**) and down then up (**h**))

Next, to further investigate the time-dependent m^6^A modification spectrum of lncRNAs, four clusters of methylation peaks (“up-and-up”, “down-and-down”, “up-then-down” and “down-then-up”, deduced from the normalized counts) were identified. As seen in the heat-maps, 212, 166, 554 and 50 lncRNA-m^6^A peaks were screened, respectively. Among them, peaks with the tendencies of “up-and-up” and “up-then-down” (with proportion of 21.6% and 56.4% in all peaks) are more than other two groups (Fig. 4a-d). Subsequently, the four types of modified lncRNA-associated mRNAs were subjected to Kyoto Encyclopedia of Genes and Genomes (KEGG) pathway analysis. Some pathways related to RNA metabolism (RNA degradation in “up-and-up” cluster, Fig. 4e), radiation injury effects (p53 signaling pathway in “down-and-down” cluster (Fig. 4f), immunity (B cell receptor signaling pathway in “up-then-down” cluster, Fig. 4g) and hematopoiesis (the only pathway of hematopoietic cell lineage in “down-then-up” cluster, Fig. 4h) were screened out. The results hint at the possible involvement of lncRNA m^6^A modification in hematopoietic radiation injuries.

### LncRNA ***Snhg15*** m^6^A modification modulates the radiation injury of BMCs

**Fig. 5:**
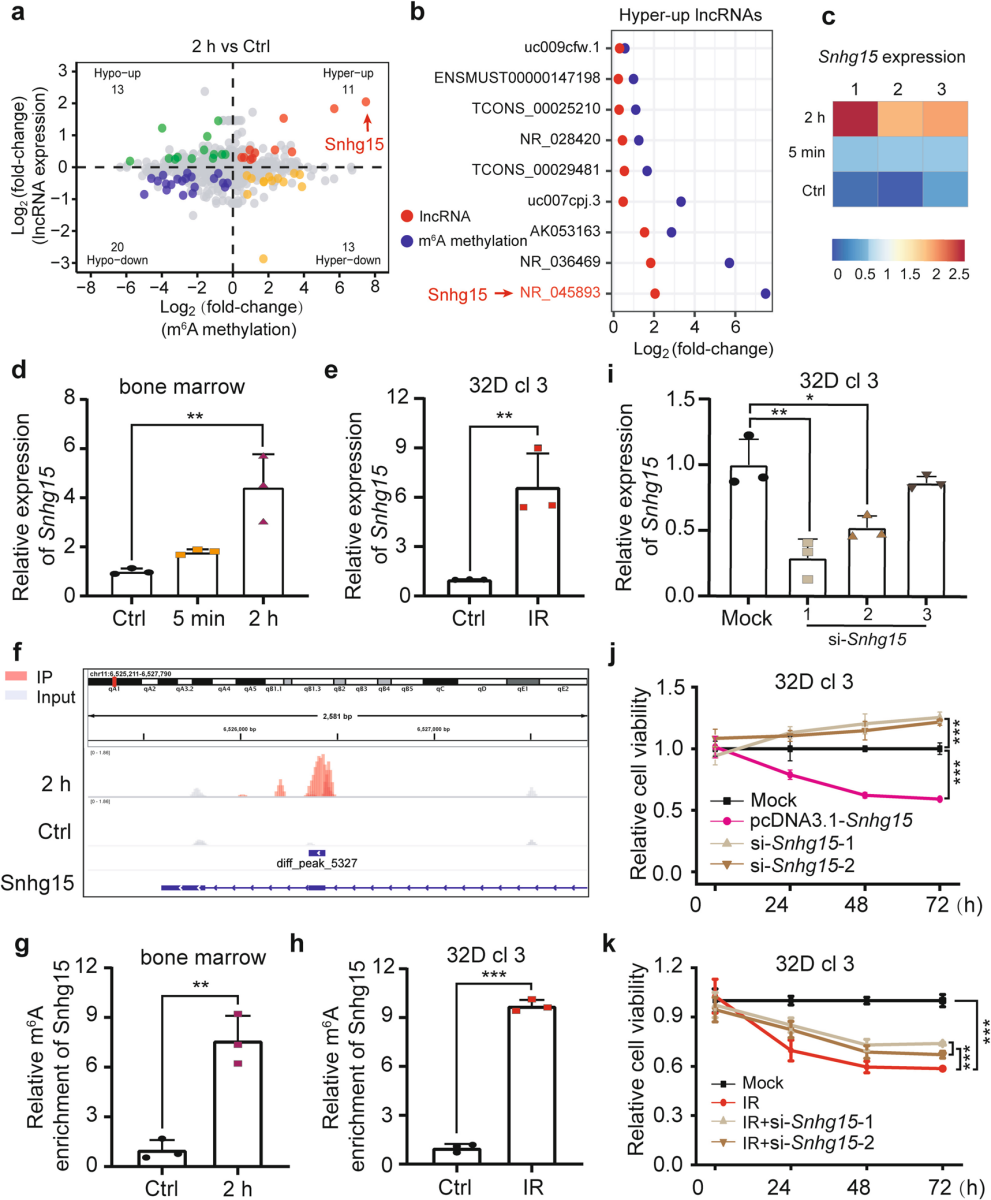
LncRNA *Snhg15* m^6^A modification modulates radiation injury of BMCs. **a** Integrated analysis of lncRNAs with differential m^6^A methylation (hyper/hypo) and transcriptional expression (up/down) between 2 h and Ctrl groups. **b** The cluster of m^6^A-modified lncRNAs of hypermethylated-upregulated (Hyper-up) trend. **c** RNA-seq heatmap showing differential *Snhg15* expression in Ctrl, 5 min, and 2 h groups. **d and e** QRT-PCR validation of *Snhg15* expression in irradiated mouse BMCs (**d**) and 32D cl 3 cells (**e**). **d**: each biological replicate pooled BMCs from 4 mice. **f** IGV visualization of *Snhg15* m^6^A methylation changes in 2 h vs Ctrl. **g** and **h** MeRIP-qPCR quantification of *Snhg15* m^6^A enrichment in irradiated BMCs (**g**) and 32D cl 3 cells (**h**). **g**: each biological replicate pooled BMCs from 4 mice. **i** Interference efficiency of three si-*Snhg15* sequences in 32D cl 3 cells. **j** and **k** CCK-8 assay measuring 32D cl 3 cell viability after *Snhg15* overexpression/knockdown (**j**) or IR/IR + *Snhg15* knockdown (**k**). (*n* = 6 biological replicates.) Data are presented as mean ± SD. Statistical significance: Student’s *t*-test for **e**, **g** and **h**; one-way ANOVA for **d** and **i-k**. *P < 0.05, **P < 0.01, ***P < 0.001

To further elucidate the mechanisms underlying the injury effects of γ-irradiation on hematopoietic cells, integration analysis of the MeRIP-seq and lncRNA transcriptomic data was performed. Particularly, given the existence of the reversible phenomena for some modifications, we focused on the result of the comparison of 2 h vs Ctrl. A series of modified-lncRNAs (with more than one significant peaks for certain lncRNAs) belonging to 4 classes were identified (Fig. 5a). Among them, 9 lncRNAs were up-regulated with hyper-methylated m^6^A modifications (11 m^6^A peaks), at 2 h after irradiation, 12 lncRNAs were down-regulated with hyper-methylated m^6^A peaks (13 m^6^A peaks), 17 lncRNAs were down-regulated with hypo-methylated m^6^A sites (20 m^6^A peaks), and 11 lncRNAs were up-regulated with hypo-methylated m^6^A decorations (13 m^6^A peaks) (Fig. 5b and Fig. S2a-c). Among these, NR_045893 (*Snhg15*), a lncRNA previously linked to injury progression [23–25], stood out due to its marked transcriptional and m^6^A methylation changes (Fig. 5a and b). RNA-seq revealed time-dependent *Snhg15* upregulation post-γ-irradiation (Fig. 5c), validated by qRT-PCR in BMCs and 32D cl 3 cells (Fig. 5d and e). IGV visualization confirmed m^6^A peaks on *Snhg15* (Fig. 5f), and MeRIP-qPCR showed increased m^6^A methylation in irradiated BMCs and 32D cl 3 cells (Fig. 5g and h).

Functional studies involved constructing a *Snhg15* overexpression vector and validating siRNA interference efficiency (Fig. 5i). Cell counting kit-8 (CCK-8) assays revealed reduced viability in *Snhg15*-overexpressing 32D cl 3 cells, whereas siRNA-treated cells displayed enhanced proliferation compared to controls (Fig. 5j). Upon irradiation, 32D cl 3 cell viability was suppressed, while further *Snhg15* knockdown rescued it to some extent (Fig. 5k). Collectively, these findings demonstrate that lncRNA *Snhg15* m^6^A modification critically regulates γ-irradiation-induced BMC injury.

### LRPPRC stabilizes ***Snhg15*** m^6^A-dependently to exacerbate radiation-elicited injury of BMCs

**Fig. 6:**
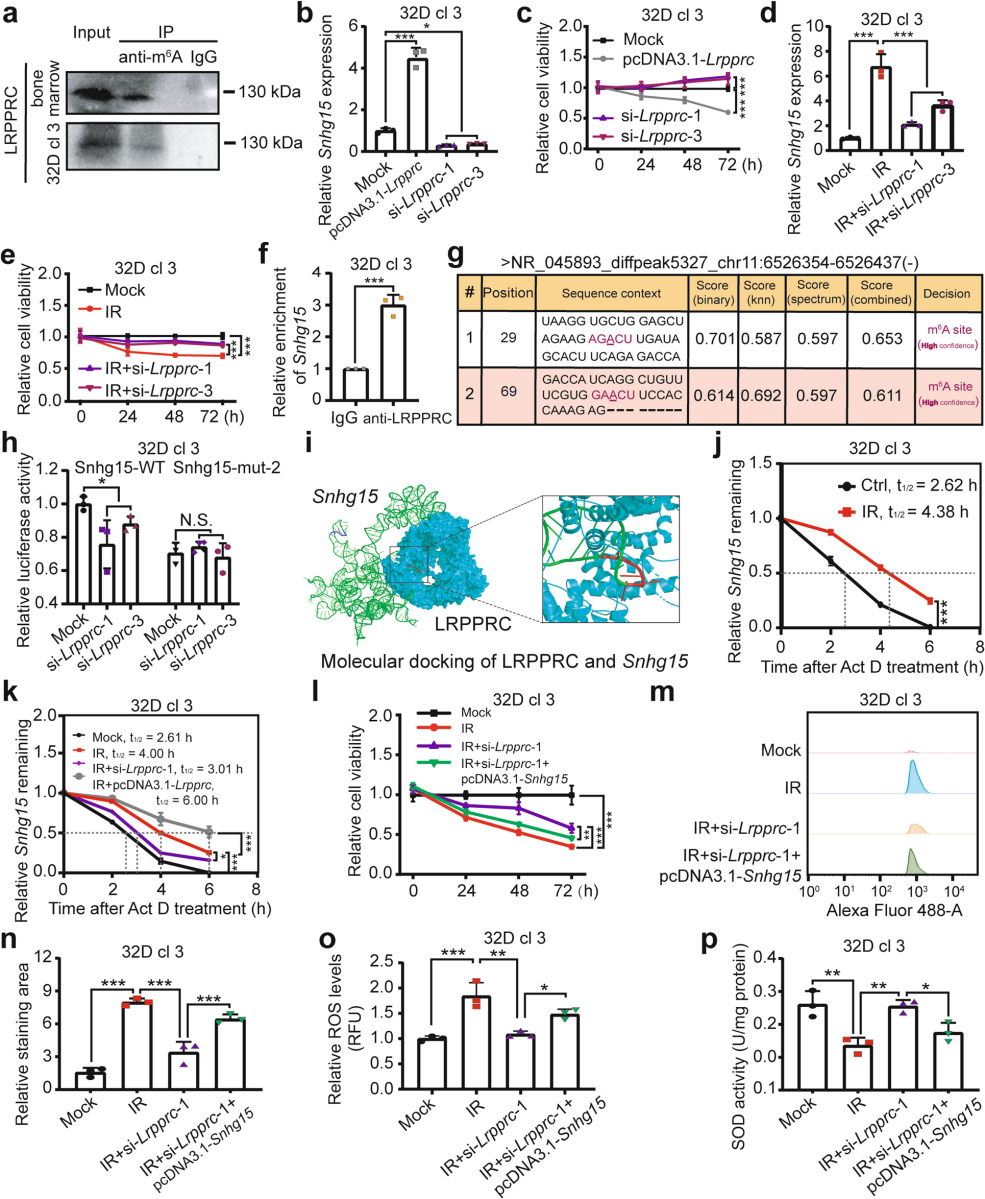
LRPPRC stabilizes *Snhg15* m^6^A-dependently to exacerbate radiation-elicited injury of BMCs. **a** Co-IP assay showing endogenous interaction between LRPPRC and m^6^A-modified RNAs in mouse BMCs and 32D cl 3 cells. **b and c** *Snhg15* expression (**b**) and cell viability (**c**, *n* = 6) following *Lrpprc* overexpression/knockdown. **d and e** *Snhg15* expression (**d**) and cell viability (**e**, *n* = 6) in irradiated 32D cl 3 cells with *Lrpprc* knockdown. **f** RIP-qPCR validation of *Snhg15* binding to LRPPRC. **g** Predicted m^6^A sites on *Snhg15* using SRAMP. **h** Dual-luciferase reporter assay of Snhg15-WT/mut-2 constructs in 32D cl 3 cells with *Lrpprc* knockdown. **i** Structural model of LRPPRC protein (blue surface) bound to *Snhg15* (green ribbon). Critical interactions (red rods) occur at the second m^6^A motif (GAACU), while the first motif (AGACU, dark blue) shows weaker binding. **j** and **k** Half-life change of *Snhg15* in irradiated 32D cl3 cells with or without *Lrpprc* manipulation. **l** CCK-8 assay measuring 32D cl3 viability in Mock, IR, IR + si-*Lrpprc-*1, and IR + si-*Lrpprc-*1 + pcDNA3.1-*Snhg15* groups. **m** and **n** Flow cytometry examining TUNEL staining (**m**) and corresponding apoptotic cell quantification (**n**). **o** and **p** ROS levels (**o**) and SOD activity (**p**) in indicated groups. Data are presented as mean ± SD. Statistical significance: Student’s *t*-test for **f **and** j**; one-way ANOVA for **b**-**e, h, k** and **l**, **n**-**p**. *P < 0.05, **P < 0.01, ***P < 0.001

The fate of m^6^A-modified RNAs is determined by Reader proteins that recognize m^6^A motifs [14, 28]. To identify the key Reader for *Snhg15* m^6^A modification and its role in γ-irradiation-induced BMC injury, we analyzed RNA-seq data revealing upregulated m^6^A Readers (e.g., *Ythdf1/2/3*, *Ythdc1*, *Igf2bp3*, *Lrpprc* and *Hnrnpc*) at 2 h post-irradiation (Fig. S3a), consistent with Fig. 1i-m and Fig. S1c. Database searches via RM2Target (http://rm2target.canceromics.org/#/detail/RM2Target_1700345) identified LRPPRC as a potential *Snhg15* Reader (Fig. S3b), supported by its emerging roles in pathological processes [16–18]. Accordingly, we focused on LRPPRC and tried to find out whether it regulates *Snhg15* as an m^6^A Reader protein in BMCs damaged by radiation. Co-immunoprecipitation (Co-IP) confirmed LRPPRC interaction with m^6^A-modified *Snhg15* in BMCs and 32D cl 3 cells (Fig. 6a). *Lrpprc* expression was induced in irradiated 32D cl 3 cells (Fig. S3c and d). Overexpression of *Lrpprc* increased *Snhg15* levels and reduced cell viability, whereas *Lrpprc* knockdown had opposite effects (Fig. S3e and f, Fig. 6b and c). Under irradiation, *Lrpprc* knockdown suppressed *Snhg15* and rescued cell viability (Fig. S3g, Fig. 6d and e). These results suggest that LRPPRC inhibits the viability of 32D cl3 through up-regulating lncRNA *Snhg15*. Next, we determined the direct interaction between *Snhg15* and LRPPRC protein. RNA immunoprecipitation (RIP) followed by qRT-PCR assay uncovered that the *Sngh15* transcript was obviously enriched by LRPPRC antibody (Fig. 6f). MeRIP-seq data showed that the m^6^A peaks of *Snhg15* located in the region of chr11: 6,526,354–6,526,437(-), and the concrete m^6^A site prediction in SRAMP website (http://www.cuilab.cn/m6asiteapp/result/9bDi8QLRCs) for this sequence revealed that there were two potential sites with high confidence (Fig. 6g and Fig. S3h). Then we constructed the luciferase reporter gene vector with the integrated sequences harboring (wild-type) WT or mutated (mut) m^6^A (the two predicted A bases within m^6^A motifs were mutated separately to C). Luciferase assay displayed that *Lrpprc* knockdown diminished the luciferase activity of Snhg15-WT vector, and the luciferase activity of Snhg15-mut-2 vector dropped but was insensitive to *Lrpprc* depletion (Fig. 6h). The luciferase activity of Snhg15-mut-1 vector displayed no significant discrepancy with that of Snhg15-WT panel (Fig. S3i). Additionally, molecular docking was performed and the highest-scoring conformation (confidence score: 0.9633) was selected for detailed analysis. *Snhg15* exhibited strong binding affinity to the cavity of LRPPRC protein. Coincidently, structural alignment revealed that GAACU motif, the second predicated modification site of *Snhg15*, rather than the first AGACU motif, formed stable contacts with conserved residues within the LRPPRC binding pocket, consistent with the luciferase reporter assay result (Fig. 6i). These data confirmed the direct interaction between LRPPRC and *Snhg15* mainly at the second m^6^A site.

Given that the known function of LRPPRC as an m^6^A Reader relates to regulating the stability of its target RNA [17, 29], we wondered whether LRPPRC up-regulated *Snhg15* through promoting its stability. First, we ascertained that IR exposure indeed prolonged the half-life of *Snhg15*. On this basis, *Lrpprc* knockdown shortened the prolonged half-life, whereas *Lrpprc* overexpression further extended it instead (Fig. 6j and k). Finally, we validated the function of LRPPRC and lncRNA *Snhg15* in γ-radiation-caused BMC injury. CCK-8 assay showed that IR-treatment suppressed the proliferation of 32 D cl 3, while transfection of si-*Lrpprc-*1 rescued it. Nevertheless, *Lrpprc* knockdown plus overexpression of *Snhg15* restrained the cell viability again (Fig. 6l). TUNEL staining revealed that *Lrpprc* interference erased the IR-caused apoptotic phenotype, but *Snhg15* overexpression in addition to *Lrpprc* depletion reversed apoptotic status (Fig. 6m and n). The levels of ROS agreed with the result of TUNEL staining among Mock, IR, IR + si-*Lrpprc-*1 and IR + si-*Lrpprc-*1 + pcDNA3.1-*Snhg15* groups. However, the contents of SOD exhibited opposite trends (Fig. 6o and p). These findings demonstrate that LRPPRC promotes the radiation injury of BMCs through *Snhg15 *in vitro. Taken together, the m^6^A Reader protein LRPPRC stabilizes lncRNA *Snhg15* in an m^6^A-dependent manner and facilitates its expression to exacerbate the BMC radiation injury.

### LRPPRC-mediated *Snhg15* up-regulation promotes radiation-induced hematopoietic injury in vivo

**Fig. 7:**
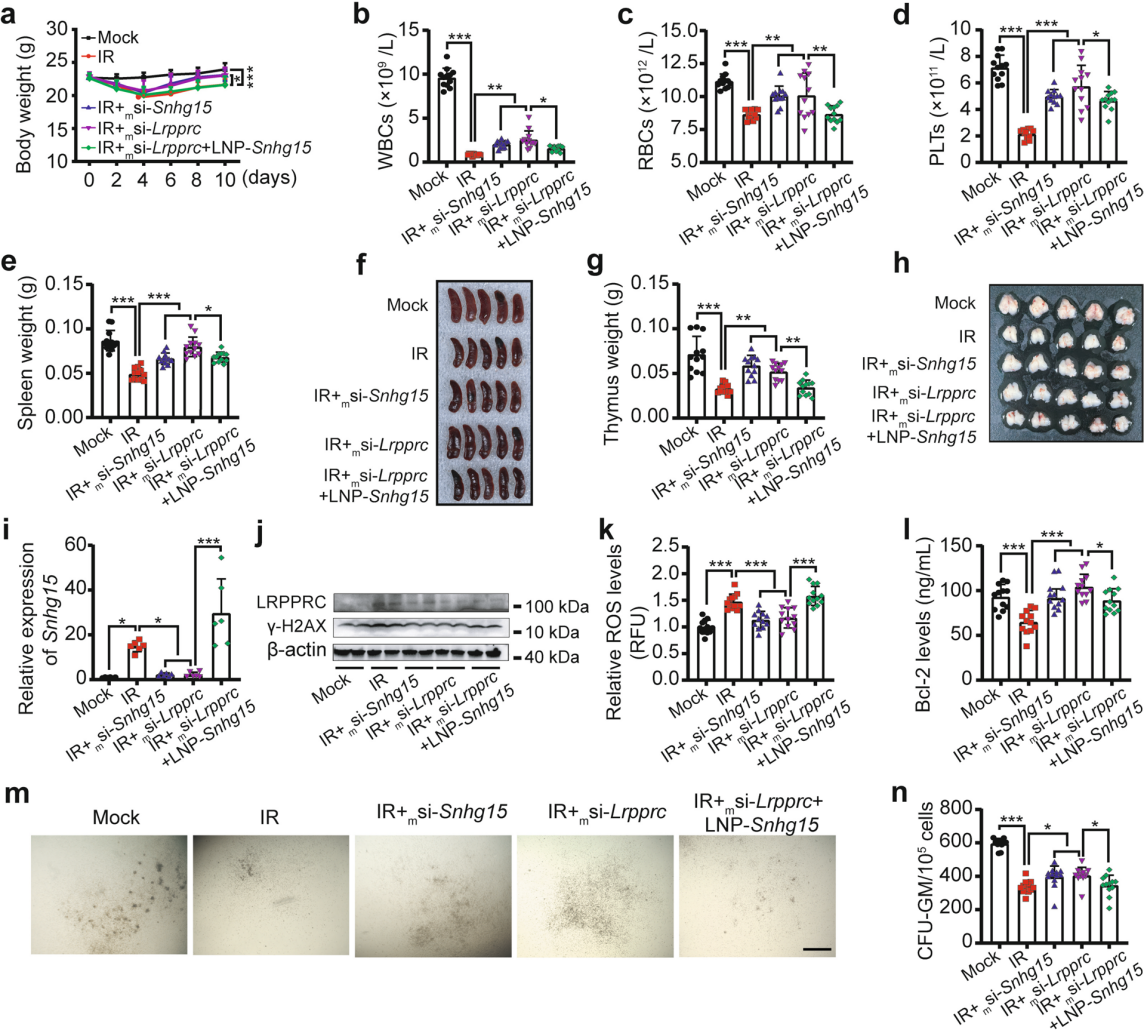
LRPPRC-mediated *Snhg15* up-regulation promotes radiation-induced hematopoietic injury in vivo. **a** Body weight changes in Mock, IR, IR + _m_si-*Snhg15*, IR + _m_si-*Lrpprc*, and IR + _m_si-*Lrpprc* + LNP-*Snhg15* groups over 10 days post-4 Gy total-body irradiation. (*n* = 12 mice/group). **b**-**d** Peripheral blood counts: white blood cells (WBCs, **b**), red blood cells (RBCs, **c**) and platelets (PLTs, **d**). **e**-**h** Spleen (**e** and **f**) and thymus (**g** and **h**) weights and representative images at the end of the experiment. **i** QRT-PCR quantification of *Snhg15* expression in BMCs. Each biological replicate pooled BMCs from 2 mice. **j** Western blot analysis of LRPPRC and γ-H2AX protein levels. Each biological replicate pooled BMCs from 6 mice. **k** and **l** ROS levels (**k**) and Bcl-2 expression (**l**) in BMCs. **m** and** n** Representative images and count statistics of CFU-GMs in each group. Scale bar: 500 μm. Data are presented as mean ± SD. Statistical significance determined by one-way ANOVA: *P < 0.05, **P < 0.01, ***P < 0.001

To investigate the roles of LRPPRC and *Snhg15* in γ-irradiation-induced hematopoietic injury in vivo, the modified animal-available siRNAs targeting *Lrpprc*/*Snhg15* (_m_si-*Lrpprc*/_m_si-*Snhg15*) and LNP-formulated pcDNA3.1-*Snhg15* (LNP-*Snhg15*) were administered via tail vein 24 h before irradiation. After a 10-day experimental period, irradiated mice showed significant weight loss compared to controls, which was partially rescued by *Snhg15*/*Lrpprc* knockdown. However, *Snhg15* overexpression in *Lrpprc*-knockdown mice reversed weight recovery (Fig. 7a). Hematological parameters [white blood cells (WBCs), red blood cells (RBCs) and platelets (PLTs)] and hematopoietic organ weights (spleen, thymus) displayed consistent trends across Mock, IR, IR + _m_si-*Snhg15*, IR + _m_si-*Lrpprc* and IR + _m_si-*Lrpprc* + LNP-*Snhg15* groups (Fig. 7b-h). *Snhg15* expression was elevated in IR and IR + _m_si-*Lrpprc* + LNP-*Snhg15* groups, correlating with injury severity (Fig. 7i). LRPPRC protein levels increased in IR and IR + _m_si-*Snhg15* groups but decreased in others (Fig. 7j). Additional injury markers mirrored these findings: γ-H2 AX and ROS levels contradicted weight/organ changes, whereas Bcl-2 and CFU-GM showed inverse trends (Fig. 7j-n). Altogether, these results demonstrate that LRPPRC-mediated *Snhg15* up-regulation exacerbates γ-radiation-induced hematopoietic injury in vivo.

### The LRPPRC-*Snhg15* axis mediates the radio-protective effects of VA on hematopoietic radiation injury

**Fig. 8:**
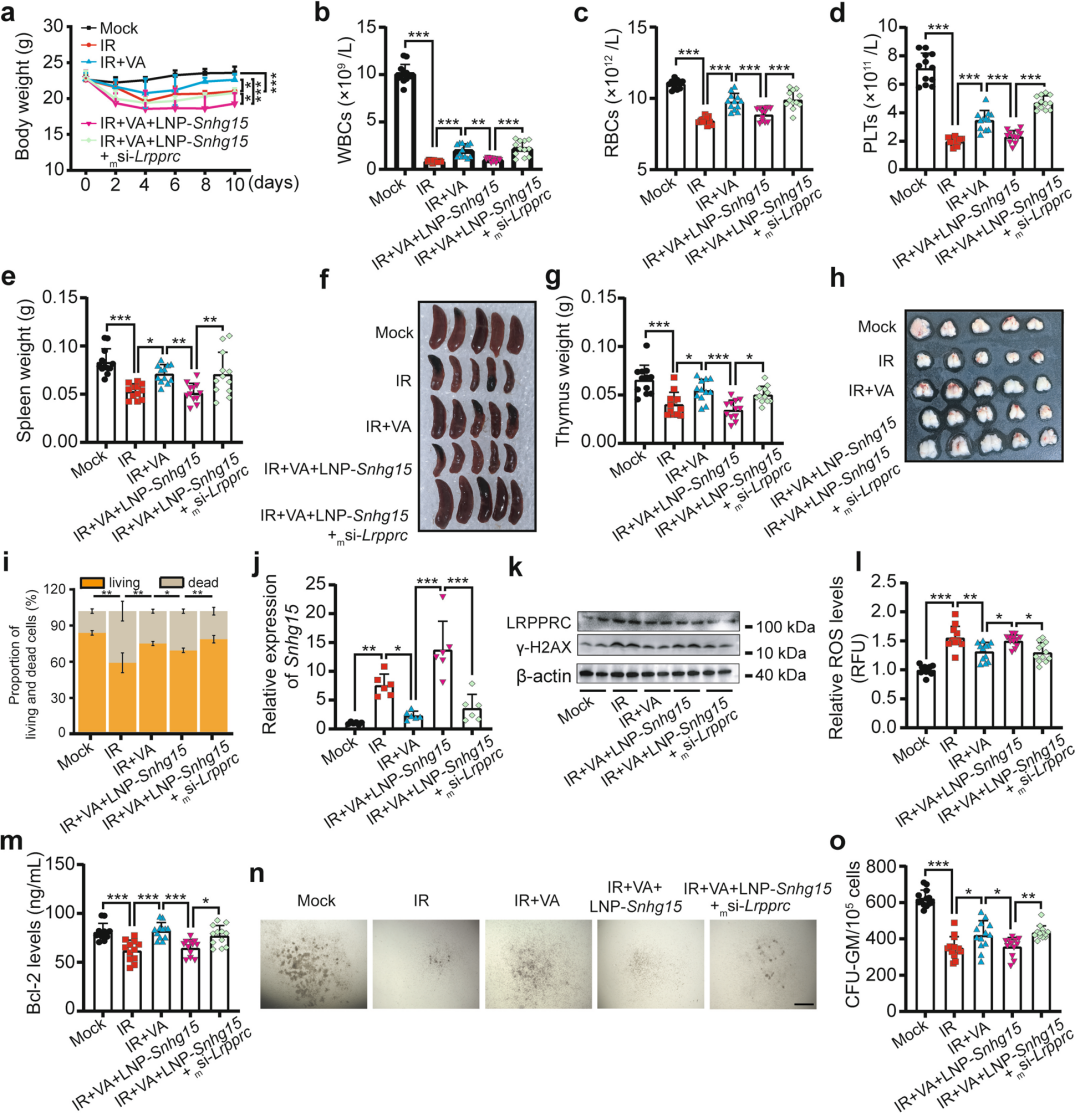
The The LRPPRC-*Snhg15* axis mediates the radio-protective effects of VA on hematopoietic radiation injury. **a** Body weight changes in Mock, IR, IR + VA, IR + VA + LNP-*Snhg15* and IR + VA + LNP-*Snhg15* + _m_si-*Lrpprc* groups over 10 days post-4 Gy TBI. (*n* = 12 mice/group). **b**-**d** Peripheral blood counts: WBCs (**b**), RBCs (**c**), and PLTs (**d**). **e**-**h** Spleen (**e** and **f**) and thymus (**g** and **h**) weights and representative images at the end of the experiment. **i** Statistical analysis of viable and dead BMC proportions. (*n* = 3 biological replicates; each replicate pooled BMCs from 4 mice). **j** QRT-PCR quantification of *Snhg15* expression in BMCs. Each biological replicate pooled BMCs from 2 mice. **k** Western blot analysis of LRPPRC and γ-H2AX protein levels Each biological replicate pooled BMCs from 6 mice. **l** and **m** ROS levels (**l**) and Bcl-2 expression (**m**) in BMCs. **n** and **o** Representative images and count statistics of CFU-GMs in each group. Data are presented as mean ± SD. Statistical significance determined by one-way ANOVA: *P < 0.05, **P < 0.01, ***P < 0.001

Our group previously reported that gut microbiota-derived VA mitigates radiation injury in hematopoietic and intestinal systems [30]. Here, we investigated whether the LRPPRC-*Snhg15* axis identified in this study contributes to VA-mediated radio-protection. In vivo experiments confirmed that VA rescued IR-induced weight loss, which was abolished by *Snhg15* overexpression but restored by *Lrpprc* knockdown (Fig. 8a). Key hematological parameters (WBCs, RBCs and PLTs) and hematopoietic organ weights mirrored these effects (Fig. 8b-h). BMC viability aligned with VA’s protective role (Fig. 8i). *Snhg15* expression positively correlated with injury severity but negatively correlated with VA efficacy (Fig. 8j). LRPPRC protein levels increased post-IR, decreased with VA treatment, and further declined with *Lrpprc* knockdown in the IR + VA + LNP-*Snhg15* group (Fig. 8k). Radiation-induced elevations of γ-H2AX and ROS were reversed by VA, reinstated by *Snhg15* overexpression, and partially restrained by *Lrpprc* knockdown (Fig. 8k and l). Bcl-2 and CFU-GM showed inverse trends (Fig. 8m-o). Collectively, these data demonstrate the LRPPRC-*Snhg15* axis mediates VA’s radio-protective effects, reaffirming its critical role in hematopoietic radiation injury.

## Discussion

Radiotherapy remains a cornerstone treatment for malignancies, yet its clinical utility is often constrained by radiation-induced damage to normal tissues. Hematopoiesis, the dynamic process responsible for generating all blood lineages, is particularly susceptible to radiation injury, representing a major dose-limiting factor in cancer treatment. This study advanced our understanding of radiation biology by investigating the roles of m^6^A modification and non-coding RNAs in γ-irradiation-induced hematopoietic injury. Our findings address three critical gaps in the field: 1) Characterizing the acute radiation response in hematopoiesis and identifying novel RNA epigenetics-based targets; 2) Unveiling the LRPPRC/m^6^A-*Snhg15* axis as a pathogenic mechanism in BMCs injury; 3) Validating gut microbiota-derived VA as an epigenetic modulator of radio-protection, aligning with the emerging “gut-blood axis” concept [31].

This study found that that γ-ray irradiation rapidly impaired bone marrow hematopoietic cells, triggering a series of events such as apoptosis, oxidative stress and DNA damage. Concurrently, the expression of m^6^A modification Reader proteins up-regulate, indicating the involvement of m^6^A modification in the early response to radiation exposure. Through m^6^A methylome and transcriptome analyses of BMC lncRNAs, it was found that the m^6^A modification of lncRNAs exhibited dynamic changes with a “change-then-recovery” trend following radiation. This trend coincides with the mRNA m^6^A fluctuating spectrum uncovered in our previous work [32], mutually confirming the reversibility of m^6^A modification and implying its potentially crucial regulatory role in the process of radiation damage. What is different is that in this work, we focused on in-depth exploration of the detailed molecular mechanism of lncRNA m^6^A modification mediating hematopoietic radiation injury, in addition to a brief description of the modification spectrum. Importantly, due to the numerical advantage of ncRNAs [33], studying the m^6^A modification on ncRNAs might be more meaningful compared to that of mRNA.

Among the numerous differentially-modified lncRNAs, *Snhg15* was identified as the candidate target because it displayed almost the utmost changes in both transcriptional expression and m⁶A methylation levels in the 2 h vs Ctrl comparison and was closely associated with the progression of radiation injury in BMCs. It has been reported that this lncRNA plays pivotal roles in regulating various injury phenotypes. For instance, it regulates the cerebral ischaemia-reperfusion injury via miR-153-3p/ATG5 axis [23]. It modulates cardiomyocyte apoptosis after hypoxia/reperfusion injury through modulating miR-188-5p/PTEN axis [24]. Its knockdown ameliorates oxygen and glucose deprivation (OGD)-induced neuronal injury via the miR-9-5p/TIPARP axis [25]. It participates in the regulation of myoblast proliferation through interacting with lncRNA Sugt1-Associated-Muscle (*SAM*) in skeletal muscle injury [26]. However, this is the first report of its role, and particularly the m^6^A modification-associated role, in radiation-induced hematopoietic injury.

Further investigation revealed that LRPPRC, as a newly discovered m^6^A Reader protein, could interact with the m^6^A modification site of *Snhg15*, stabilize *Snhg15* and promote its expression, thereby exacerbating radiation injury to BMCs. In vivo experiments also confirmed that the LRPPRC-*Snhg15* signaling pathway was involved in γ-ray irradiation-induced hematopoietic injury. LRPPRC, initially identified as a protein involved in mitochondrial function and energy metabolism [15], has recently been reported to function as an m^6^A Reader. In ischemic stroke, it has been found that LRPPRC is one of the key regulators of m^6^A and serves as an immune marker. This explains the role of LRPPRC in immune metabolism, which may be related to m^6^A modification regulating immune related genes [19]. One of the main functions of m^6^A Readers is to regulate the stability of modified RNAs. For example, in triple-negative breast cancer (TNBC), it was found that LRPPRC could bind to the m^6^A-modified mRNA of lactate dehydrogenase A (*LDHA*). This binding protected *LDHA* mRNA from degradation, resulting in the enhanced glycolysis in TNBC cells and promoting tumor cell growth and invasion [17]. LRPPRC aggravates inflammation to promote malignancy of breast cancer cell through increasing the m^6^A modification of C-X-C motif chemokine ligand 11 (*CXCL11*) [34]. In this study, we revealed that LRPPRC stabilizes lncRNA *Snhg15* in an m^6^A-dependent manner, which enriches the evidence that LRPPRC acts as m^6^A Reader protein to regulate the stability of lncRNAs.

It is noteworthy that this study also explored the relationship between the LRPPRC-*Snhg15* axis and the protective effect of gut microbiota-derived VA against hematopoietic radiation injury. It was found that VA could mitigate the damage to the body weight and hematopoietic system of mice caused by radiation. However, overexpression of *Snhg15* could disrupt this protective effect, and further interfering with *Lrpprc* could partially restore it. This indicates that the LRPPRC-*Snhg15* signaling pathway plays an important role in the radio-protective function of VA and further validates the critical significance of this signaling axis in the progression of hematopoietic radiation injury. This work reconfirms the “crosstalk” between gut microbiome and the hematopoietic system, thus verifying the significance of the “gut-blood axis” [35, 36] and providing new ideas for the target-based development of novel radio-protective agents.

m^6^A modification exerts precise regulation over the expression of genes implicated in injury or disease progression, which highlights its potential as both diagnostic biomarkers and therapeutic targets and underscores its translational value in precision medicine [37]. Given the radiosensitivity of hematopoiesis [38], targeting the LRPPRC-*Snhg15* axis may offer therapeutic benefits for radiotherapy patients and radiation accident victims. Strategies could include small molecule inhibitors of LRPPRC/*Snhg15* or gene-editing approaches to alter *Snhg15* m^6^A modification.

While this study establishes the LRPPRC-*Snhg15* axis in hematopoietic radiation injury, downstream effector mechanisms remain incompletely characterized. Future work should: elucidate the full regulatory network of LRPPRC-*Snhg15*, including potential microRNA/protein interactors; investigate additional m^6^A-modified ncRNAs and Readers involved in radiation responses; and validate the VA-LRPPRC-*Snhg15* axis in translational models to inform clinical development. By bridging epigenetic mechanisms, gut metabolites, and hematopoietic injury, this study provides a novel framework for developing targeted radio-protective strategies.

## Conclusion

This study uncovers a novel mechanism where the m^6^A-modified lncRNA *Snhg15*, stabilized by the m^6^A Reader protein LRPPRC, exacerbates γ-irradiation-induced hematopoietic injury. Our findings highlight the critical role of lncRNA m^6^A modification in radiobiology and provide a mechanistic framework for understanding acute radiation responses. By identifying the LRPPRC-*Snhg15* axis, this work establishes novel potential therapeutic targets for mitigating radiation-induced hematopoietic damage. Additionally, our discovery of VA-mediated regulation of this axis underscores the translational potential of gut microbiota metabolites in radio-protection. Collectively, these results advance RNA epigenetics research in radiation biology and inspire the development of m^6^A-targeted interventions to alleviate radiotherapy side effects.

## Materials and methods

### Experimental animals

Male C57BL/6 J mice (6–8 weeks old, body weight: 20 ± 2 g) were purchased from HFK Bioscience (Beijing, China) and housed in a specific-pathogen-free (SPF) animal facility at the Institute of Radiation Medicine (IRM), Chinese Academy of Medical Sciences (CAMS). Mice were maintained under standard conditions (ambient temperature: 22 ± 2 °C, relative humidity: 40 ~ 70%, 12 h light/12 h dark cycle) with ad libitum access to rodent chow and sterile water. The mice were acclimatized to the housing conditions for 1 week before the experiment. They were randomly assigned into groups (*n* = 12 per group) for different experimental conditions.

### Cell culture and transfection

The mouse hematopoietic 32D clone 3 (32D cl 3) cell line was purchased from biobw Biotechnology Co., LTD (bio-69708, Beijing, China) and cultured in RPMI-1640 medium (11,875,093, Gibco, CA, USA) supplemented with 10% fetal bovine serum (FBS, P1020-500, Gibco), 10 ng/mL mouse interleukin-3 (IL-3, PMC0035, Gibco) at 37 °C in a humidified atmosphere containing 5% CO₂. For transfection, 2 × 10^5^ or 5 × 10^3^ cells were seeded in 6- or 96-well plates for 24 h and transfected with 1 μg/well (6-well) or 50 ng/well (96-well) plasmid DNA, or 50 nmol/L (6-well) or 5 nmol/L (96-well) siRNA using Lipofectamine® 2000 reagent (11,668,019, Invitrogen, CA, USA) according to the manufacturer’s protocol.

### Plasmid construction and siRNA synthesis

The lncRNA *Snhg15* overexpression plasmid was synthesized by inserting the sequence of *Snhg15* (NR_045893) into the pcDNA3.1 vector at the NheI and XhoI sites, termed as pcDNA3.1-*Snhg15*. The fragment of > NR_045893_diffpeak5327_chr11:6,526,354-6,526,437 (-): GAA ACC TAA GGT GCT GGA GCT AGA AGA GAC TTG ATA GCA CTT CAG AGA CCA TCA GGC TGT TTC GTG GAA CTT CCA CCA AAG AG containing the predicted high confidential m^6^A sites (underlined) of *Snhg15* was synthesized and inserted into the pmirGLO vector to generate the Snhg15-WT luciferase reporter construct. Two mutant fragments of this sequence carrying a single substitution of the predictably modified A with C was synthesized to construct Snhg15-mut-1 and Snhg15-mut-2 as above. In in vivo experiments, the plasmid as well as the empty vector was wrapped into the lipid nanoparticle (LNP) material in a 1:3 mass ratio to obtain the LNP-*Snhg15* or LNP-pcDNA3.1 plasmid. SiRNAs targeting *Snhg15* or *Lrpprc*, termed as si-*Snhg15* or si-*Lrpprc* and their respective negative controls were synthesized. Their sequences are listed in Supplementary Table S1. SiRNAs, along with negative controls, were synthesized by GeneCreate Biotechnology Co., Ltd. (Jiangsu, China). For in vivo studies, siRNAs were modified with 2'-O-methyl (2'-OMe) and 5'-cholesterol (_m_si-*Snhg15*/_m_si-*Lrpprc*) to enhance stability.

### Irradiation study

Irradiation was performed using a Gammacell® 40 ^137^Cs Exactor (Atomic Energy of Canada Ltd., Canada) at a dose rate of 0.84 Gy/min. Mice received a single 4 Gy TBI or sham irradiation. Post-irradiation, mice were euthanized immediately (0 h) or at 5 min/2 h for BMC collection (*n* = 12/group). For in vitro experiments, 32D cl3 cells were irradiated with 4 Gy in culture plates. Sham-irradiated cells were incubated at similar temperatures for the same duration.

### In Vivo experimental groups

(1) For investigation of the immediate effect of γ-irradiation on BMCs and sample collection for MeRIP-seq or RNA-seq, mice were randomly divided into 3 groups: Control (Ctrl), 5 min and 2 h. These animals were exposed to 4 Gy of TBI or sham irradiation, followed by the rapid euthanasia at 0, 5 min and 2 h post-irrradiation for BMC collection. (*n* = 12). (2) For in vivo investigation of the role of *Snhg15* and *Lrpprc* in hematopoietic radiation injury, mice were randomly divided into 5 groups: Mock, IR, IR + _m_si-*Snhg15*, IR + _m_si-*Lrpprc* and IR + _m_si-*Lrpprc* + LNP-*Snhg15*. _m_siRNAs (10 nmol/mouse, 100 μL) and LNP-*Snhg15* (20 μg/mouse, 200 μL), as well as the negative control small interfering RNAs or corresponding empty plasmid, were administered via tail vein every 3 days, starting 24 h before irradiation. The study lasted 10 days with weighing the mice every other day. (3) For in vivo investigations of the role of the LRPPRC-*Snhg15* axis in the radio-protective efficacy of VA, the animals were separated randomly into 5 groups: Mock, IR, IR + VA, IR + VA + LNP-*Snhg15*, IR + VA + LNP-*Snhg15* + _m_si-*Lrpprc*. The in vivo siRNA and plasmid injections were manipulated as described in (2) following the experimental settings of each group. Mice receiving VA treatment were administrated with VA (109-52-4, Aladdin, Shanghai, China) solution (0.3 mg/ml, 200 μL/mouse) [30] through oral route every day, initiating from 1 h before irradiation. The mock group was gavaged with equal volume of distilled water.

### Western blot and Co-IP

Total protein lysates were obtained from mouse BMCs or 32D cl 3 cells using RIPA (R0010, Solarbio, Beijing, China) buffer following the manufacturer’s procedures. 1 × 10^7^ cells were added to 1 mL of RIPA. Proteins were separated through 12% sodium dodecyl sulfate-polyacrylamide gel electrophoresis (SDS-PAGE). The gel was transferred to a polyvinylidene fluoride membrane (IPVH00010, Millipore, MA, USA). After blocking, antibodies specific for γ-H2AX (9718, CST, USA), YTHDF1 (17479-1-AP, Proteintech, Wuhan, China), YTHDC1 (14392-1-AP, Proteintech, Wuhan, China), IGF2BP3 (81805-1-RR, Proteintech Wuhan, China), LRPPRC (21175-1-AP, Proteintech, Wuhan, China) and HNRNPC (MA5-32270, Thermo Fisher Scientific, California, USA) were used. The membrane was incubated with anti-β-actin (66009-1-1 g, Proteintech, Wuhan, China) as an internal control. The HRP-labelled secondary antibodies (GB23301, Servicebio, China and SA00001-2, Proteintech, Wuhan, China) were used. Reads were detected using ChemiDoc XRS + and Image Lab software (Version 5.1, Bio-Rad, Shanghai, China). A co-IP kit (22202, Beaverbio, Jiangsu, China) was used according to the manufacturer’s procedures. Mouse BMCs or 32D cl 3 cells were harvested into the IP buffer, followed by a centrifugation at 12, 000 rpm for 15 min to obtain the supernatant. The anti-m^6^A antibody (4 μg, ab208577, Abcam, Cambridge, United Kingdom)-coupled Protein A/G beads (22202, Beaverbio, Jiangsu, China) were added to the supernatant and incubated at 4℃ overnight. Next, the denatured IP samples were subjected to immunoblotting with the LRPPRC antibody. IgG (B900620, Proteintech, Wuhan, China) was used here as a negative control.

### Identification of methylation regions and detection of differential methylation regions

The raw sequencing reads were first trimmed using the Cutadapt software (version 4.1) [39] to obtain clean reads. Subsequently, these clean reads were aligned to the mouse reference genome (mm10) using the HISAT2 software (version 2.2.1) [40]. Enriched methylation regions were identified between each MeRIP-seq sample and the corresponding input control sample using the MACS2 software (version 2.2.7.1) [41], with default parameters employed. Finally, differential methylation regions (DMRs) between the two groups were detected using the DiffReps software (version 1.55.6) [42]. A region was defined as a DMR if it met the criteria of |Log2(Fold Change)|≥ 5 and *P*-value < 1 × 10^⁻10^.

### Motif analysis

We selected the top 5000 methylated regions that showed the highest fold-enrichment from the MACS2 results. Subsequently, we used the bedtools software (version 2.30.2) [43] to extract the corresponding genomic sequences. Then, we performed motif analysis on these sequences using the STREME software (version 5.5.7), [44] to identify potential regulatory elements.

### Integration analysis

We combined the differential methylation table and the differential expression table for the comparison between the 2 h group and the control group based on the Transcript_ID. A scatter plot was generated using ggplot2 with a difference threshold of *p* < 0.05. We averaged the log2 (fold-change) of the four types of peaks in the integration analysis according to the Transcript_ID, and then plotted a dot plot using the ggplot software. The Integrative Genomics Viewer (IGV) software (version 2.16.0) [45] was used to visualize the differentially methylated peaks between the two groups.

### Statistical analysis

We analyzed the data using appropriate statistical methods and presented the results as the mean ± standard deviation (SD) for each group, with the sample size denoted as (n). We used one-way analysis of variance (ANOVA) to assess statistical significance among multiple groups and Student’s* t*-test to analyze statistical significance between two independent groups. The significant differences were denoted as follows: *, *P* < 0.05; **, *P* < 0.01; ***, *P* < 0.001.

## Supplementary Information

Supplementary Material 1

## Data Availability

The MeRIP- and RNA- sequencing data have been deposited in the National Genomics Data Center, China National Center for Bioinformation (CNCB-NGDC) database (https://ngdc.cncb.ac.cn/, accession number: CRA025623). The research data generated or analyzed during this study are included in this published article and its Supplementary Material. Other data from the corresponding authors are available upon reasonable request.
