# Supplementary material for: Leucine-rich pentatricopeptide repeat-containing protein (LRPPRC)-stabilized lncRNA small nucleolar RNA host gene 15 (Snhg15) modulates hematopoietic injury induced by γ-ray irradiation via m6A modification
Source: Mol Biomed. 2025 Jun 25;6:44. doi: 10.1186/s43556-025-00279-2 (PMC12187624; doi:10.1186/s43556-025-00279-2)
Supplement: Supplementary file 1 — Supplementary Material 1 [file 43556_2025_279_MOESM1_ESM.docx]

**Leucine-rich pentatricopeptide repeat-containing protein (LRPPRC)-stabilized lncRNA small nucleolar RNA host gene 15 (*Snhg15*) modulates hematopoietic injury induced by** **γ-ray irradiation *via* m^6^A modification**

Shuqin Zhang *, Yajia Cheng, Yujia Gao, Feifei Xu, Yuna Wang, Junling Zhang, Yue Shang, Deguan Li, Saijun Fan *

*State Key Laboratory of Advanced Medical Materials and Devices, Tianjin Key Laboratory of Radiation Medicine and Molecular Nuclear Medicine, Tianjin Institutes of Health Science, Institute of Radiation Medicine, Chinese Academy of Medical Sciences & Peking Union Medical College, Tianjin, 300192, People’s Republic of China.*

*** Corresponding authors:**

Shuqin Zhang: zhangshuqin@irm-cams.ac.cn

Saijun Fan: [fansaijun@](mailto:fansaijun@irm-cams.ac.cn)[irm-cams.ac.cn](mailto:fansaijun@irm-cams.ac.cn)

Shuqin Zhang and Yajia Cheng contributed equally to this work.

**Supplementary figures and figure legends**

**
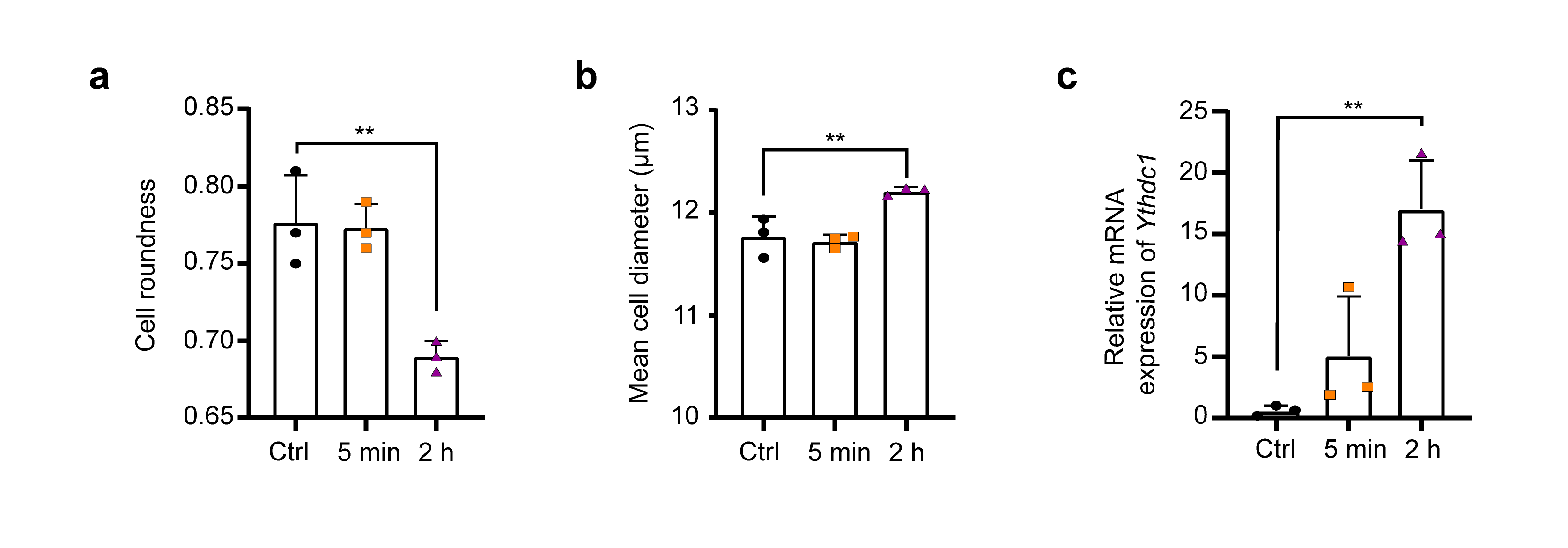
**

**Fig. S1** **Rapid damage of bone marrow hematopoietic cells by γ**-**ray irradiation involves altered expression of m^6^A Readers.** **a and b** The statistical analysis of the roundness (**a**) and mean diameter (**b**) of bone marrow cells in Ctrl, 5 min and 2 h groups. Each biological replicate pooled BMCs from 4 mice. **c** QRT-PCR analysis of m^6^A Reader YTH domain containing 1 (*Ythdc1)* mRNA expression. Each biological replicate pooled BMCs from 4 mice. Data are presented as mean ± SD. Statistical significance was determined by one-way ANOVA: **P* < 0.05, ***P* < 0.01.

**
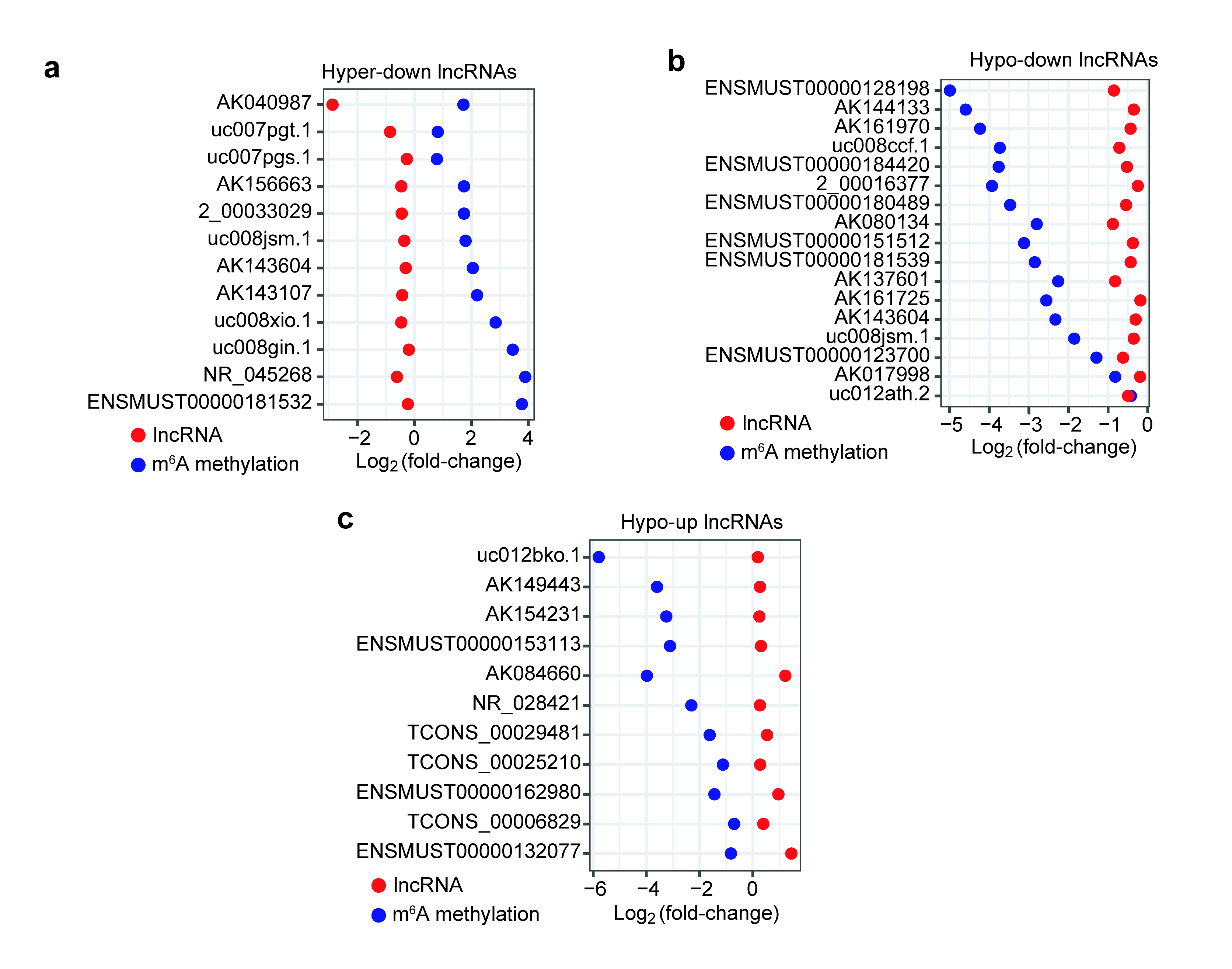
**

**Fig. S2 The clusters of m^6^A**-**modified lncRNAs.** **a-c** hypermethylated-downregulated (Hyper-down, **a**), hypomethylated-downregulated (Hypo-down, **b**) and hypomethylated-upregulated (Hypo-up, **c**).

**
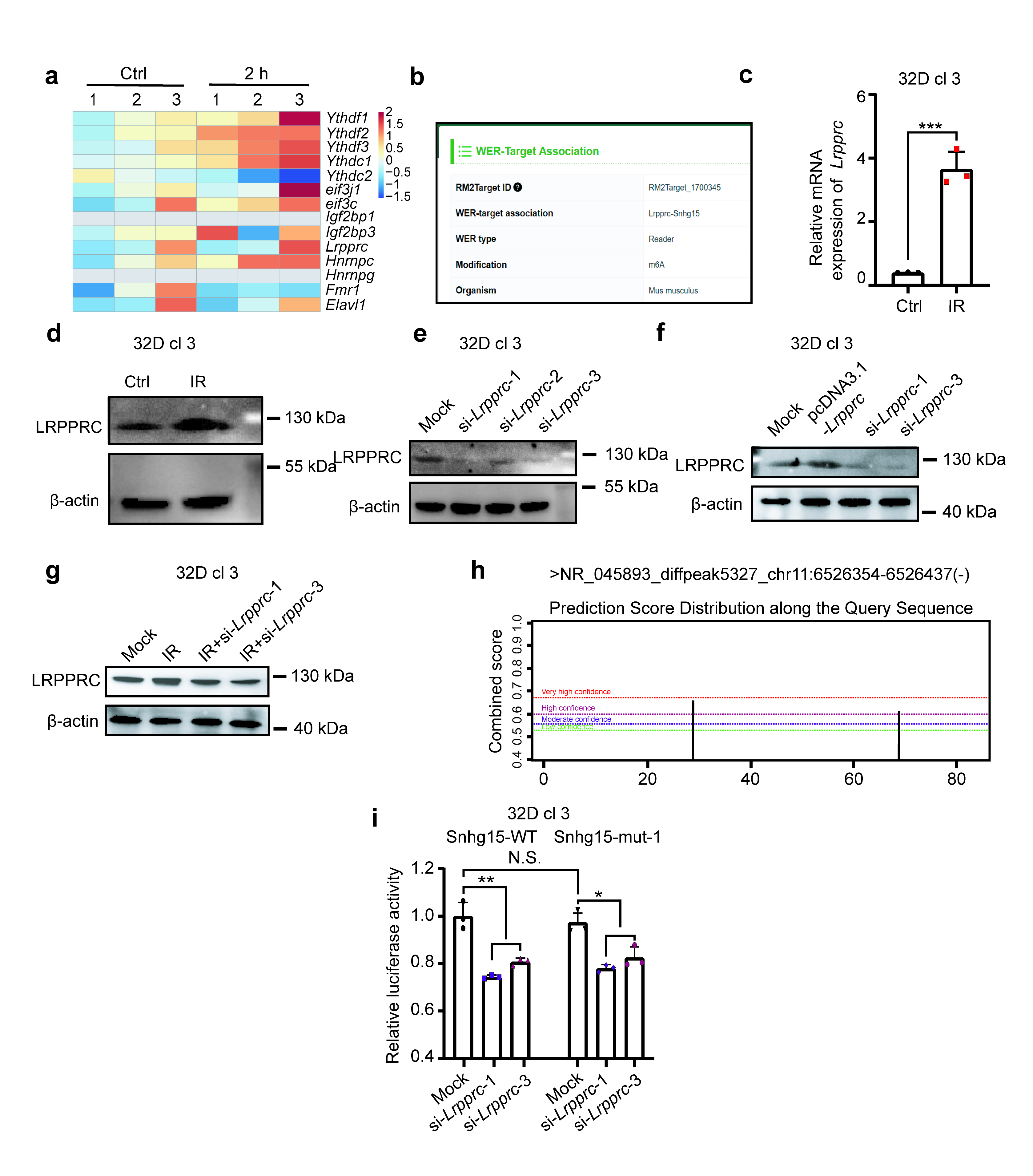
**

**Fig. S3 LRPPRC stabilizes *Snhg15* m^6^A**-**dependently to exacerbate radiation**-**elicited injury of BMCs. a** The heatmap showing the expressions of common m^6^A Reader proteins in BMCs of Ctrl and 2 h groups, from the RNA-seq data. **b** Prediction of the m^6^A Reader protein LRPPRC recognizing and binding to lncRNA *Snhg15*. **c and d** QRT-PCR (**c**) and Western blot (**d**) analysis of *Lrpprc* expression in irradiated 32D cl 3 cells. **e** Western blot assay examining the interfering efficiency of *Lrpprc* siRNAs in 32D cl 3 cells. **f** The protein expression levels of *Lrpprc* in 32D cl 3 were detected by Western blot assay following overexpression or knockdown of *Lrpprc*. **g** The protein expression levels of *Lrpprc* in 32D cl 3 were detected by Western blot assay after the treatment of IR or IR plus *Lrpprc* interference. **h** The schematic showing the prediction of m^6^A modification status were obtained using SRAMP website. **i** Dual-luciferase reporter vectors with the wild type (WT) or mutant (mut-1) m^6^A nucleotide in *Snhg15* modification region were transfected into 32D cl 3 cells with or without *Lrpprc* interference. Then the luciferase activity was measured. Data are presented as mean ± SD. Statistical significance: Student’s *t*-test for **c**; one-way ANOVA for **i**: **P* < 0.05, ***P* < 0.01, ****P* < 0.001.

**Supplementary materials and methods**

**Peripheral blood cell count**

Peripheral blood was collected from the orbital sinus of mice into the tubes containing the anti-coagulant EDTA, which was mixed thoroughly to prevent coagulation. The hemogram indexes covering white blood cells (WBCs), red blood cells (RBCs) and platelets (PLTs) were measured using a fully automated haemocytometer (MEK-1305, Nihon kohden, Tokyo, Japan).

**Measurement of spleen, thymus and bone marrow cell (BMC) survival**

At the end of the experiment, the spleens and thymuses of euthanized mice were dissected for weight measurement and photographing. BMCs flushed from the marrow lumen were filtered (2 mL), diluted 10 folds and counted using a Countstar automated cell counter (IE1000, KEYENCE, Osaka, Japan), with the simultaneous statistics of the proportion of living and dead cells.

**Cell counting kit-8 (CCK-8) assay**

Cell viability was assayed using CCK-8 (K1018, APExBIO, Huston, USA). Briefly, 32D cl 3 cells were inoculated onto 96-well plate (2000/well) for 24 h and then were transfected, followed by the irradiation 24 h post-transfection. 10 μL/well of CCK-8 reagent was added after 0, 24, 48 and 72 h of irradiation, respectively, followed by 2 hours’ incubation. The absorbance at 450 nm was measured using a microplate reader (RT-6500, Rayto, Shenzhen, China).

**Enzyme-linked immunosorbent assay (ELISA)**

For quantification of Cysteine aspartate-specific proteinase-3 (Caspase-3) and B-cell lymphoma/Leukemia-2 (Bcl-2) in BMCs, corresponding ELISA kits (TW10333 and TW8425, Tongwei, Shanghai, China) were used according to the manufacturer’s instructions. Briefly, BMCs were rinsed with pre-cooled PBS, then the precipitates were obtained by centrifugation, ground thoroughly (0.1 g tissue or 1×10^7^ cells in 900 μL PBS) on ice and centrifuged again. The protein concentration of the supernatant was determined using a spectrophotometer. The supernatant was added into the antibody-coated wells of the microplate, then the Enzymeconjugate were supplemented to react in 37℃ for 1 h. The microplate was washed for absorbance measurement, and the optical density was read at 450 nm (RT-6500, Rayto, Shenzhen, China). The ultimate levels were normalized to the protein concentration.

**Determination of the levels of oxidative stress-related markers**

The supernatants were prepared as described in the ELISA Part. The sample was prepared and added into the microplate of the commercial reactive oxygen species (ROS) and superoxide dismutase (SOD) detection kits (G0163W and G0101W, Grace Biotechnology, Suzhou, China) according to the manufacturer’s instructions. Then, it was incubated at 37℃ in the dark for 30 min at room temperature. For ROS detection, the fluorescence value was read at the excitation wavelength of 488 nm and emission wavelength of 525 nm using EnSpire Multimode Plate Reader (PerkinElmer, MA, USA). For SOD detection, the absorbance was determined at a wavelength of 450 nm. The ultimate levels of the two markers were normalized to the protein concentration.

**Colony forming unit of granulocyte macrophage cell (CFU-GM) assay**

Approximately 2×10^4^ BMCs from the unirradiated group and 2×10^5^ BMCs from the irradiated groups were added to the 24-well plate in methylcellulose medium (M3234, STEMCELL Technologies, Vancouver, Canada). The plate was incubated in 37℃, 5% CO_2_ for 5 days, followed by observation, imaging and counting the number of colonies with more than 30 cells.

**Terminal deoxynucleotidyl transferase-mediated deoxyuridine triphosphate-nick end labelling (TUNEL) Staining**

BMCs were washed with PBS and then were detected for the apoptosis status using the One Step TUNEL Apoptosis Assay Kit (C1088, Beyotime Biotechnology, Shanghai, China) according to the manufacturer’s instructions. A single sample of 2×10^6^ cells was collected. TUNEL assay solution was added to incubate at 37 ºC for 1 h in dark. After washing, cells were suspended in PBS and detected by flow cytometry FACS (Celesta, BD, New Jersey, USA) with excitation wavelength range of 450~500 nm and emission wavelength range of 515~565 nm.

**Dual-luciferase reporter assays**

The 32D cl 3 cells were inoculated onto the 96-well plate at a density of 5×10^3^ cells per well, and were transiently co-transfected with the plasmids of Snhg15-WT, Snhg15-mut-1 or Snhg15-mut-2 and siRNAs of *Lrpprc* (si-*Lrpprc*-1 or si-*Lrpprc*-3) as well as the negative control siRNA. After 48 h, the luciferase reporter gene assay was performed using the Dual-Luciferase Reporter Assay System (F6075M, Uelandy, Suzhou, China) according to the manufacturer’s instructions. The absorbance was determined using EnSpire Multimode Plate Reader (PerkinElmer, MA, USA). The Renilla luciferase activity contained in pmirGLO vector was used as the internal normalization.

**Quantitative real-time polymerase chain reaction (****qRT-PCR)**

Total RNA was extracted from mouse BMCs or 32D cl 3 cells using RNAiso Plus (9109, T akara, Tokyo, Japan) according to the manufacturer’s protocol. 1 mL RNAiso Plus was added per 10^7^ cells. Reverse-transcribed was performed by Hifair^®^ Ⅱ 1st Strand cDNA Synthesis Kit (11149ES60, YEASEN, Shanghai, China). qRT-PCR was performed according to the instructions of Fast Start Universal SYBR Green Master (Rox) (04913914001, Roche Diagnostics GmbH, Mannheim, Germany) as described previously [[1](#_ENREF_1)]. Relative fold changes were calculated as 2^-ΔΔCt^, and β-actin was used for internal normalization. The experiments were performed in triplicate in three independent assays. All primers are listed in Supplementary Table S2.

**MeRIP-qPCR**

The m^6^A modification levels of each lncRNA were detected using Magna MeRIP^TM^ m^6^A kit (17-10499, Millipore, MA, USA) according to the manufacturer’s instructions. In brief, 4 μg of total RNA was extracted from mouse BMCs or 32D cl 3 cell line, and the RNA was fragmented into approximate 100 nt-long sequences which were immunoprecipitated with m^6^A antibody (ab208577, Abcam, Cambridge, United Kingdom)- or mouse IgG-conjugated beads at 4℃ overnight. The precipitated RNA was eluted and then recovered. One tenth of the RNA samples were retained as Input. Enrichment of m^6^A-lncRNA was monitored by qRT-PCR and normalized to 10-fold of Input. The primers used for each lncRNA are listed in Supplementary Table S2.

**LncRNA *Snhg15* stability assay**

32D cl 3 cells with corresponding treatments were treated with actinomycin D (5 μg/mL, A432787, Aladdin, Shanghai, China) to disrupt the global RNA transcription. Then, the cells were harvested at 0, 2, 4 and 6 h post-treatment, followed by total RNA extraction and *Snhg15* qRT-PCR assay.

**RNA immunoprecipitation (RIP)-qPCR assay**

The RIP assay was performed according to the instructions of PureBinding^®^ RNA Immunoprecipitation Kit (P0402, Geneseed, Guangzhou, China). In brief, 1×10⁷ 32D cl 3 cells were harvested into 1 mL lysis buffer. 5 μg LRPPRC antibody and the IgG control was conjugated to protein A/G beads, and the beads were incubated with the cell lysate in RIP buffer overnight at 4℃, and were then resuspended in PBS. The DNA was removed and RNA was purified for qRT-PCR assay.

**Molecular docking**

Molecular docking simulations were conducted to explore the interaction between LRPPRC and *Snhg15* using the HDOCK server (version 2.0; http://hdock.phys.hust.edu.cn/) following established protocols [[2](#_ENREF_2)]. The secondary structure of *Snhg15* was initially predicted *via* RNA Fold software, and its tertiary structure was modeled using the 3dRNA program (http://biophy.hust.edu.cn/new/3dRNA). Model quality was assessed by the integrated 3dRNAscore module, with the optimal model (score: 26.3284) selected for docking. To address the long-chain complexity of *Snhg15*, a segmented prediction approach combined with the “Multi-Chain” mode in HDOCK was implemented. The LRPPRC crystal structure (UniProt ID: AF-Q6PB66-F1) was retrieved from the UniProt database and preprocessed using MOE’s Protein Preparation Wizard (version 2022.02). Missing residues were reconstructed using the QuickPDB database and Loop Modeler tool (AMBER10 force field, 100 sampling iterations). Protonation states were optimized with the Protonate 3D module (pH 7.4, 0.15 M salt concentration) to reflect physiological conditions. A blind docking strategy was employed with an 80 Å×80 Å×80 Å grid box encompassing potential binding regions. Initial rigid-body global sampling generated 10,000 conformations, which were clustered (RMSD cutoff=7.0 Å) and refined to yield the top 100 models. The 10 lowest-energy conformations (ranked by HDOCK score) were analyzed using PyMOL (version 2.5; https://pymol.org/2/) for alignment with the receptor’s binding site and identification of key interacting residues. This protocol ensured reproducibility and structural insights into the Lrpprc-Snhg15 interaction mechanism.

**MeRIP-seq library construction and sequencing**

BMCs from every four mice in the same group were made into a mixture to obtain three replicates per group. Subsequently, total RNA was extracted using TRIzol reagent (Invitrogen Corporation, CA, USA) according to the manufacturer’s instructions. The Ribo-Zero rRNA Removal Kit (Illumina, Inc., CA, USA) was employed to deplete ribosomal RNA. The RNA was then chemically fragmented into approximately 100-nucleotide fragments using a fragmentation buffer (Illumina, Inc.).

MeRIP-Seq was carried out by Cloudseq Biotech, Inc. (Shanghai, China) with slight adjustments to a previously reported protocol [[3](#_ENREF_3), [4](#_ENREF_4)]. Briefly, RNA fragments were incubated with an anti-m^6^A polyclonal antibody (202003; Synaptic Systems, Göttingen, Germany) in IP buffer at 4°C for 2 h and then immunoprecipitated using protein-A beads (Thermo Fisher Scientific, MA, USA) at 4°C for 2 h. Next, the m^6^A-modified RNA (BERRY & ASSOCIATES, PR3732, MI, USA) coupled to the beads was eluted in IP buffer, followed by extraction with TRIzol reagent (Thermo Fisher Scientific). The purified RNA was collected for RNA-seq library construction using the NEBNext Ultra™ RNA Library Prep Kit (New England Biolabs, MA, USA). Both m^6^A IP samples and non-IP input samples were subjected to 150-bp paired-end sequencing on an Illumina HiSeq 4000 sequencer (Illumina, Inc.).

**Peak annotation**

MACS2 enriched methylated peak were annotated to lncRNA genes based on Ensembl GTF annotation database (v87). Additionally, the distribution of peaks within different regions (5'END, 33%, 66% and 3'END) was determined. ClusterProfiler R packages were used to perform GO and KEGG pathway enrichment analysis based on the associated mRNAs of m^6^A peak-related lncRNAs [[5](#_ENREF_5)].

**References**

1. Zhang SQ, Yang Z, Cai XL, Zhao M, Sun MM, Li J, et al. miR-511 promotes the proliferation of human hepatoma cells by targeting the 3'UTR of B cell translocation gene 1 (BTG1) mRNA. Acta Pharmacol Sin. 2017;38(8):1161-70. <https://doi.org/10.1038/aps.2017.62>.

2. Yan Y, Tao H, He J, Huang SY. The HDOCK server for integrated protein-protein docking. Nat Protoc. 2020;15(5):1829-52. <https://doi.org/10.1038/s41596-020-0312-x>.

3. Luo Z, Zhang Z, Tai L, Zhang L, Sun Z, Zhou L. Comprehensive analysis of differences of N(6)-methyladenosine RNA methylomes between high-fat-fed and normal mouse livers. Epigenomics. 2019;11(11):1267-82. <https://doi.org/10.2217/epi-2019-0009>.

4. Meyer KD, Saletore Y, Zumbo P, Elemento O, Mason CE, Jaffrey SR. Comprehensive analysis of mRNA methylation reveals enrichment in 3' UTRs and near stop codons. Cell. 2012;149(7):1635-46. <https://doi.org/10.1016/j.cell.2012.05.003>.

5. Yu G, Wang LG, Han Y, He QY. clusterProfiler: an R package for comparing biological themes among gene clusters. OMICS. 2012;16(5):284-7. <https://doi.org/10.1089/omi.2011.0118>.

**Supplemental Table S1.** List of sequences of siRNAs used in this study.

| **Gene** | **Primer** | | **Sequence (5**'-**3**'**)** | |
| --- | --- | --- | --- | --- |
| si-*Lrpprc*-1 | | sense strand | GGGAGAUGACGAUGCUCUATT | |
|  |  | antisense strand | UAGAGCAUCGUCAUCUCCCTT | |
| si-*Lrpprc*-2 | | sense strand | GGACAGAGUUUGCUCACAATT | |
|  |  | antisense strand | UUGUGAGCAAACUCUGUCCTT | |
| si-*Lrpprc*-3 | | sense strand | GCAGGAGAUGAAUGUUAAATT | |
|  |  | antisense strand | UUUAACAUUCAUCUCCUGCTT | |
| si-*Snhg15*-1 | | sense strand | GGCUCACAAUUAUCUGUAATT | |
|  |  | antisense strand | UUACAGAUAAUUGUGAGCCTT | |
| si-*Snhg15*-2 | | sense strand | GAGACUUGAUAGCACUUCATT | |
|  |  | antisense strand | UGAAGUGCUAUCAAGUCUCTT | |
| si-*Snhg15*-3 | | sense strand | GGCUCACAAUUAUCUGUAATT | |
|  |  | antisense strand | UUACAGAUAAUUGUGAGCCTT | |
| si-negative control | | sense strand | UUCUCCGAACGUGUCACGUTT | |
|  |  | antisense strand | ACGUGACACGUUCGGAGAATT | |

**Supplementary Table S2.** List of primers used in this paper.

| **Gene** | **Primer** | | | **Sequence (5**'-**3**'**)** | |  |
| --- | --- | --- | --- | --- | --- | --- |
| *Snhg15* | | | forward | CTGGCGGGTCCTTCCTAGC | |  |
|  |  |  | reverse | ATCTCTACCTGGACTCGGAATAGC | |  |
| *Snhg15*  (MeRIP-qPCR) | | | forward | GGTGCTGGAGCTAGAAGAGAC | |  |
|  |  |  | reverse | CTCTTTGGTGGAAGTTCCACG | |  |
| *Lrpprc* | | | forward | GCTGGACTTGACACGATTGGACTC | |  |
|  |  |  | reverse | GGTTCTCGGAAGCAAGCAGGTG | |  |
| *Ythdf1* | | | forward | CCCTGTCCTGGAGAAACTGAAAGC | |  |
|  |  |  | reverse | GTACTTGATGGAGCGGTGGATGTC | |  |
| *Igf2bp3* | | | forward | CATCTGTTTATTCCCGCCCTGTCC | |  |
|  |  |  | reverse | TCACCATCCGCACTTTAGCATCTG | |  |
| *Hnrnpc* | | | forward | GCCAAAAGTGAACCGAGGAAAAGC | |  |
|  |  |  | reverse | GCAATAGGAGGAGGAGGAGGAACC | |  |
| *Ythdc1* | | | forward | GCAAGCAGATCCAGCCAGTCTTC | |  |
|  |  |  | reverse | CCCCTCCTTCCTCCTCATTCTCAG | |  |
| ENSMUST00000160174 | | | forward | TGGTCACAGCTCTAGAGTTCT | |  |
|  |  |  | reverse | TTGTCCCACGGTTCTCAGAG | |  |
| ENSMUST00000146068 | | | forward | TCTGCCCATTTAGAGGACTGT | |  |
|  |  |  | reverse | CCACACAGCAAGCAGATCAA | |  |
| ENSMUST00000172017 | | forward | | | ACATCTTCAGTCACGAGCGA | |
|  |  | reverse | | | TCATCAGTACCGTTCCCCAG | |
| NR_003633 | | forward | | | GGGCGTTTATCATCCTTGGG | |
|  |  | reverse | | | CAGCATTCTTCTCCGCATCC | |
| uc007brw.1 | | forward | | | GCCCTTCTCTCGTCACATGA | |
|  |  | reverse | | | GCTTGGGGAGACTAGAGCAC | |
| uc009ivz.1 | | forward | | | AGAAGCTTGGTGGTGAGGAA | |
|  |  | reverse | | | TCTGTGGCCTGATGTGAAGT | |
| *Actb* | | forward | | | TTCTTTGCAGCTCCTTCGTT | |
|  |  | reverse | | | ATGGAGGGGAATACAGCCC | |
